# Weak Compliance Undermines the Success of No-Take Zones in a Large Government-Controlled Marine Protected Area

**DOI:** 10.1371/journal.pone.0050074

**Published:** 2012-11-30

**Authors:** Stuart J. Campbell, Andrew S. Hoey, Jeffrey Maynard, Tasrif Kartawijaya, Joshua Cinner, Nicholas A. J. Graham, Andrew H. Baird

**Affiliations:** 1 Marine Program, Wildlife Conservation Society, Bogor, West Java, Indonesia; 2 ARC Centre of Excellence for Coral Reef Studies, James Cook University, Townsville, Queensland, Australia; 3 Red Sea Research Center, King Abdullah University of Science and Technology, Thuwal, Kingdom of Saudi Arabia; 4 Le Centre de Recherches Insulaires et Observatoire de l'Environment, Ecole Pratique des Hautes Etudes, Moorea, Polynesie Francaise; University of Western Australia, Australia

## Abstract

The effectiveness of marine protected areas depends largely on whether people comply with the rules. We quantified temporal changes in benthic composition, reef fish biomass, and fishing effort among marine park zones (including no-take areas) to assess levels of compliance following the 2005 rezoning of the government-controlled Karimunjawa National Park (KNP), Indonesia. Four years after the rezoning awareness of fishing regulations was high amongst local fishers, ranging from 79.5±7.9 (SE) % for spatial restrictions to 97.7±1.2% for bans on the use of poisons. Despite this high awareness and strong compliance with gear restrictions, compliance with spatial restrictions was weak. In the four years following the rezoning reef fish biomass declined across all zones within KNP, with >50% reduction within the no-take Core and Protection Zones. These declines were primarily driven by decreases in the biomass of groups targeted by local fishers; planktivores, herbivores, piscivores, and invertivores. These declines in fish biomass were not driven by changes in habitat quality; coral cover increased in all zones, possibly as a result of a shift in fishing gears from those which can damage reefs (i.e., nets) to those which cause little direct damage (i.e., handlines and spears). Direct observations of fishing activities in 2009 revealed there was limited variation in fishing effort between zones in which fishing was allowed or prohibited. The apparent willingness of the KNP communities to comply with gear restrictions, but not spatial restrictions is difficult to explain and highlights the complexities of the social and economic dynamics that influence the ecological success of marine protected areas. Clearly the increased and high awareness of fishery restrictions following the rezoning is a positive step. The challenge now is to understand and foster the conditions that may facilitate compliance with spatial restrictions within KNP and marine parks worldwide.

## Introduction

Throughout much of the tropics, the continued provision of goods and services from marine habitats is threatened by escalating human population densities, habitat degradation, destructive fishing methods, and increased market access and mobility of fishing fleets (e.g., [1]–[6]). The resultant degradation of marine ecosystems has greatly increased the need for effective resource management [7], [8]. Marine protected areas (MPAs) are seen as a key management and conservation tool to halt or reverse declines in habitat and fisheries resources [9]–[12]. It is not surprising therefore, that the establishment of MPAs is on the rise [13], [14].

In Indonesia, the geographic extent of reefs and reef area is greater than in any other country [15]. Approximately 60% of Indonesia's population of 240 million lives within 50 km of the coast. This, coupled with human population growth, limited employment opportunities, dwindling land area and quality for agriculture, and the open access to fisheries has led to increased dependence on coastal marine resources This increased dependence on coastal marine resources is being compounded by a range of global (e.g., climate change) and local stressors (e.g., pollution, destructive fishing practices, coastal development) that are threatening the productivity of these systems (e.g., [16]–[19]). Given these threats, improved management is necessary to ensure the sustainability of Indonesia's marine resources now and into the future.

Indonesia currently has eight marine national parks (Bunaken, Karimunjawa, Kepulauan Seribu, Kepulauan Togean, Laut Sawu, Takabonerate, Teluk Cenderawasih, and Wakatobi National Parks) encompassing approximately 10 million hectares of coastal marine habitat [20], [21]. Other Indonesian national parks, while not marine national parks by designation, also encompass significant marine habitat (most notably Komodo National Park [22]). Following the signing of the Convention on Biodiversity in 1994, the Indonesian government signaled their intention to double the area of marine waters within MPAs by 2020, have designated areas closed to fishing (i.e., no-take zones) within all MPAs, and to review zoning plans every five years [23]. Together with these marine national parks there is national legislation that prohibits the use of destructive fishing gears, including explosives, poisons, muroami and large trawl nets that damage coral reef habitats [24], [25]. This commitment to expand no-take zones and review zoning plans on a regular basis is a positive step. However, few studies have explicitly assessed whether no-take zones in existing MPAs in Indonesia are achieving their primary goal of protecting fish populations (but see [26], [27]), and to date, none cover a timeframe of even five years.

The Karimunjawa National Park (KNP) was designated as a Strict Natural Reserve in 1986, formally legislated as the Karimunjawa National Park in 1988 with the first zoning plan being released in 1989 [28], [29]. Encompassing over 100, 000 hectares of marine habitat it is the 6^th^ largest of Indonesia's marine national parks. The original zoning involved negligible input from local communities and stakeholders, and zones either prohibited all forms of fishing or allowed traditional fishing activities only. The inception of the KNP resulted in more effective compliance of local fishers with national laws prohibiting the use of destructive fishing techniques (particularly explosives) and the harvest of iconic marine species (namely turtles, dolphins, whales), however compliance with spatial restrictions was limited [30], [31]. In 2005 the KNP was rezoned incorporating community and stakeholder input to include no-take areas, areas allowing for specific activities such as tourism and aquaculture, and traditional fisheries. There was extensive community consultation during the rezoning process [26]. Although the total area to be included within each of the zones was prescribed by the government, the communities through self-organised village planning groups had considerable influence on the locations of the respective zones. The village planning groups consisted of individuals elected by the village community (as opposed to hand-picked individuals with connections to people in power [21]) to represent them at government and stakeholder meetings outside the village. The village planning groups also liaised with government and other stakeholders, and organized community consultation meetings. These meetings were open to all members of the community and were communicated to the villages several weeks in advance, thereby allowing everyone to attend and provide input into the rezoning process.

The objective of this study, therefore, was to assess the compliance with the 2005 rezoning of the KNP and determine how effective the rezoning was in protecting fish communities. Specifically, we quantify temporal changes in benthic composition, biomass of major fish functional groups and fishing effort among zones inside the park and open access sites outside the park for four years following the 2005 rezoning of the KNP. We also quantify the level of resource dependency and awareness of restrictions among local communities, and investigate the frequency of patrols and records of enforcement.

## Methods

### Study site

**Figure 1:**
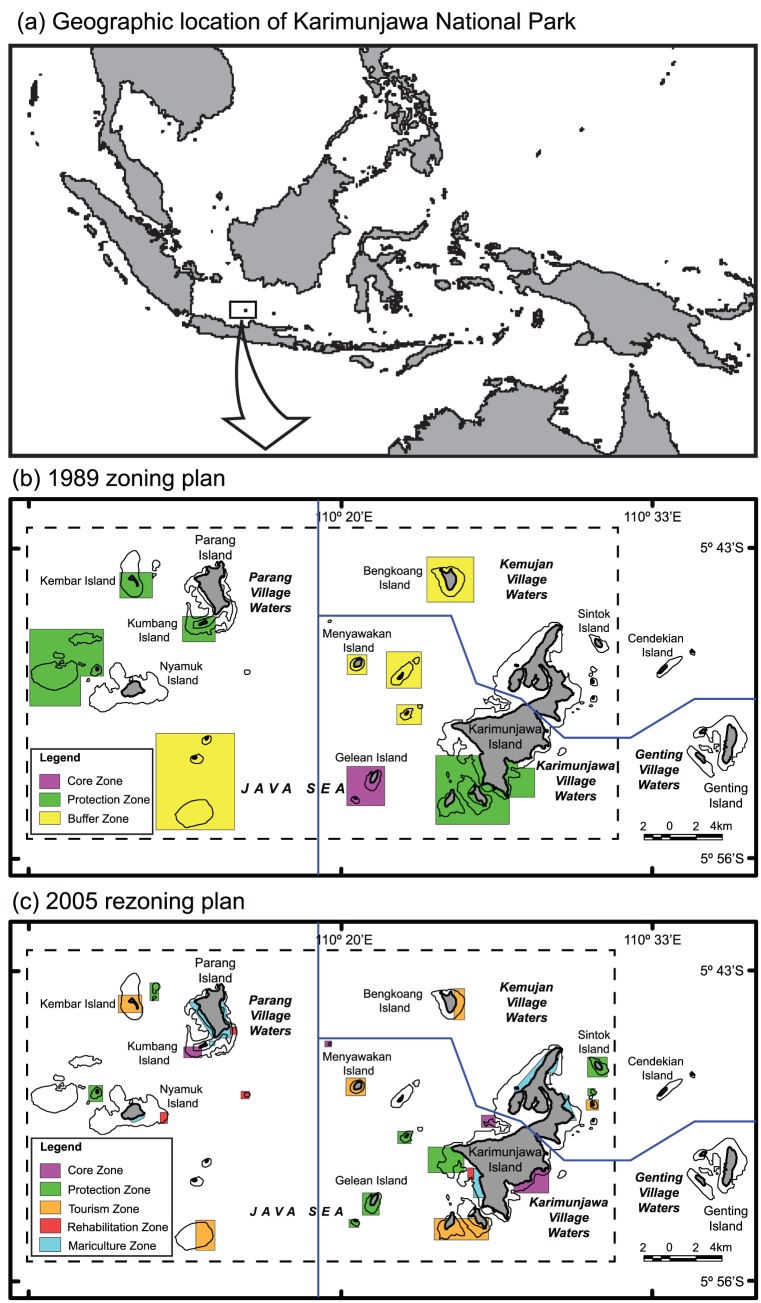
Map of Karimunjawa National Park, Indonesia. **A.** Map of Indonesia showing geographic location of Karimunjawa National Park. **B.** Map of Karimunjawa National Park showing the 1989 zoning plan. **C.** Map of Karimunjawa National Park showing the 2005 zoning plan that was legislated mid-2005. In total the park contains 22 islands and the surrounding marine environments fall within the different zone designations. Core and Protection zones are no-take zones, the distinction being that entry is permitted in Protection zones. Utilisation zones are the marine waters within the KNP boundary that are not contained within any of the other management zones.

Karimunjawa National Park (KNP) is situated 120 km north of Semarang in Central Java, Indonesia and covers 111,625 ha (Figure 1a). The Karimunjawa Islands is made up of 27 individual islands, 22 of which are located within the KNP. The islands are within one district, or Kabupaten (Jepara district, Karimunjawa sub-district), with approximately 9,000 people living in the villages (Desa) of Karimunjawa, Kemujan, Parang, and Nyamuk. Each village is composed of one to nine sub-villages, or Dusun (Karimunjawa: 9 Dusun; Kemujan: 4 Dusun; Parang: 2 Dusun; Nyamuk: 1 Dusun). Genting, the eastern islands outside the KNP boundaries (Figure 1), is a Dusun within Karimunjawa Desa. The communities are governed by leaders of their respective Desa and Dusun; each Desa has a village head, and each Dusun has a leader, or Kamituwo. Zonation within the park is based on a pre-determined set of legally prescribed zones legislated for Marine National Parks in Indonesia. In 1989 the zoning for allowable use of marine resources in KNP was legislated by the Park Authority with negligible stakeholder input, and included no-take Core and Protection Zones where all forms of fishing were prohibited, and Buffer and Utilisation Zones in which traditional fishing activities were permitted (Figure 1b). The distinction between the two no-take zones is that entry into Core zones is prohibited, while entry is permitted in Protection zones. In 2005 a new set of zones were legislated incorporating community and stakeholder knowledge and needs. These included the no-take Core and Protection Zones, ‘traditional’ fishing Utilisation Zones and zones that permitted specific activities, namely Tourism Zones, Rehabilitation Zones, and Mariculture Zones (Figure 1c). Although the total area within no-take zones (i.e., Core and Protection Zones) decreased following the rezoning, the number of no-take zones and the area of coral reef habitat within these zones increased (Table 1, Figure 1). This was due to large areas of non-reef habitat (i.e., oceanic waters) being included within no-take areas of the 1989 zoning plan.

**Table 1:** Changes in the size and number of areas within each management zones following the 2005 rezoning of the Karimunjawa National Park.

		Karimunjawa Village		Kemujan Village		Parang Village		TOTAL	
		1989	2005	1989	2005	1989	2005	1989	2005
Core	area (ha)	874.9	678.8				111.2	874.9	790.0
	no.	1	3				1	1	4
Protection	area (ha)	3,505.7	900.3		257.2	3,900.4	222.3	7,406.1	1,379.8
	no.	1	4		2	3	2	4	8
Buffer	area (ha)	1,229.3		1,238.6		4,464.8		6,932.6	
	no.	3		1		1		5	
Toursim	area (ha)		873.8		268.0		577.4		1,719.3
	no.		2		2		2		6
Mariculture	area (ha)		89.6		309.8		296.1		695.4
	no.		1		3		3		7
Rehabilitation	area (ha)		33.8		20.6		98.2		152.7
	no.		1		1		3		5
Utilisation	area (ha)	24,815.7	27,849.7	23,122.3	23,505.3	45,521.3	52,581.3	93,459.4	103,936.3

The size (ha) and number of areas within each zones are given for the three main villages, both independently and collectively. Number of areas within the utilisation zone are not given as this zone encompasses all marine waters within the Karimunjawa National Park boundary that are not contained within any of the other management zones.

### Biological surveys

Benthic and fish assemblages were quantified using underwater visual censuses within each of the KNP zones during March–April in 2005, 2006, and 2009. Additional fish surveys were conducted at the same sites within all zones during March–April 2007. In each sampling period surveys were conducted within both shallow (2–4 m) and deep (6–8 m) habitats within each of 2–11 sites within each zone (Core: 8–11 sites; Protection: 11 sites; Tourism: 2–7 sites; Utilisation: 10 sites) and 4 open access sites outside the KNP. Details of the sampling effort and locations of each site are given in Table S1. Surveys were not conducted within the Mariculture Zone as these were located in nearshore waters with limited coral habitat. The cover of live coral and algae was quantified using four replicate 50-m point-intercept transects at each depth within each site. Any live scleractinian (hard) coral or algae directly under 100 points spaced at 50 cm intervals were recorded.

Species-level surveys of all non-cryptic reef fishes (excluding pomacentrids) were conducted using underwater visual census along two to four 50-m belt transects within each depth at each site. One diver recorded all fishes greater than 10 cm total length (TL) in a 5-m wide belt, while a second diver recorded all fishes less than 10 cm TL in a 2-m wide belt. To account for differences in transect widths (2 or 5 m) fish densities were standardized to individuals per hectare, and converted to biomass using published length-weight relationships for each species, following [32]. To allow comparisons among trophic groups each fish species was categorized as a coralivore, herbivore, benthic invertivore, piscivore, planktivore, detritivore or omnivore based on diet [33]. Details of the fish species recorded and their trpohic groupings are given in Table S2.

### Fishing effort

To examine the effect of the rezoning of the KNP on the distribution of fishing activities, landing site surveys (following [31]) were conducted on Karimunjawa Island immediately prior to and 4-years after the legislation of the zoning regulations (i.e., 2004–5 and 2009–10 respectively). Sampling effort focused on Karimunjawa Island as the majority of fish caught in the region are landed on this island. A team of surveyors monitored five major fish landing sites over 14 monthly sampling periods during both 2004–5 and 2009–10 recording the number of fishing trips, fishing gear used, and interviewed the fishers to determine the zone in which fish were caught (i.e., stated compliance). Observations and discussions with local fishers suggest that the proportion of unsuccessful fishing trips was low (<5%), however this was difficult to quantify as boats that did not catch fish rarely returned to the landing sites. Consequently, only successful trips (i.e., those that landed fish) were included in the landing site surveys of fishing activities (hereafter fishing impact). While In total, 219 days were sampled during 2004–5 (mean = 15.6±1.4 SE days.month^−1^), and 230 days sampled during 2009–10 (mean = 16.4±0.8 days.month^−1^). Fishing impact was standardized to the mean number of fishing trips per day for each month.

Direct observations of fishing activities (hereafter observed fishing trips) in each of the KNP zones were used to supplement the fishing data derived from fish landing sites. Fishing activities were quantified at each of 30 sites (Core: 4 sites; Protection: 8 sites; Tourism: 6 sites; Utilisation: 12 sites) during two days of each month in 2009. Sites were visited by boat and the exact location of any vessels actively fishing was recorded. The order in which sites were visited, and consequently the route between sites, was randomised among surveys. ArcGIS was used to determine the area of fishing habitat at each of the 30 sites, and fishing effort was expressed as fishing trips km^−2^ d^−1^. While this measure of observed fishing trips is likely to underestimate total fishing effort, it does provide a relative measure and facilitate comparisons among management zones.

### Resource dependency

To assess the level of dependency of marine resources household surveys were conducted prior to the rezoning in the three main communities within the KNP: Karimunjawa (n = 96), Parang (n = 45), and Kemujan (n = 38). Households were systematically sampled (i.e., sampling every second or third house, see [34] for comprehensive methodological details), and the number of households surveyed within each community was proportional to community size. The surveys targeted the head of the household; however, in some situations, clarification about livelihood activities was sought from other household members. Respondents were asked to list the primary activities that household members engaged in to bring food or money into their house.

### Awareness of park regulations, patrolling and enforcement

To determine whether levels of awareness of fishing restrictions among fishers changed after the 2005 rezoning, surveys of fisher households were conducted in early 2005 and 2009: Karimunjawa (2005: n = 87, 2009: n = 67), Parang (2005: n = 48, 2009: n = 39), and Kemujan (2005: n = 26, 2009: n = 44). These households were systematically sampled, and the head fisher of each household was surveyed on their awareness of fishing restrictions in the park (i.e., spatial closures, species restrictions, bans on the use of poisons and explosives). Specifically, fishers were asked: (i) Are there any areas where they are not supposed to fish? (ii) Are there any species you are not supposed to catch? (iii) Are there any fishing gears you are not supposed to use? If the fishers answered ‘yes’ to any of these questions they were then asked to name or describe the area, species, and/or gear/s relating to these restrictions. Fishers were considered to be aware of the respective restriction if they successfully described the area, species, or gear. Additionally, park rangers were interviewed and government records reviewed to determine the frequency of patrolling, the number of fines levied and cases prosecuted for violators of spatial closures, species restrictions, and gear restrictions.

### Statistical analyses

Variation in the cover of live coral and algae, and the biomass of fishes, both collectively and the five major trophic groups independently (i.e., coralivores, herbivores, benthic invertivores, piscivores, planktivores) was compared among years, management zones and sites using a series of nested 3-factor ANOVAs. The low biomass of detritivores and omnivores precluded analyses for these groups. Separate analyses were conducted for shallow and deep habitats. Year and management zone were fixed orthogonal factors, and site was nested within management zone. Assumptions of the ANOVA were examined by residual analysis. Subsequently the cover of live coral and algae were arcsin-square root transformed, and fish biomass was log transformed.

Estimates of fishing impact from fish landings were compared among years and management zones using a 2-factor ANOVA, with year and management zone fixed factors. Variation in the use of fishing gear types were compared among years and management zones using a 2-factor MANOVA. Finally, observed fishing trips were compared among zones using a one-way ANOVA. Fishing impact data were log-transformed to meet the assumptions of the analyses.

Changes in the awareness of fishing restrictions (i.e., spatial closures, species restrictions, and bans on the use of explosives, nets and poisons) were compared before and after the rezoning (i.e., 2005 vs 2009) using chi square tests.

### Ethics statement

The activities for this study were conducted under permission from the Indonesian government. The Wildlife Conservation Society (WCS) has a Memorandum of Understanding with the Indonesian Ministry of Forestry and Conservation, and a Technical Agreement with the Karimunjawa National Park Authority that allows work towards conservation goals and marine conservation in Indonesia. No fauna or flora were collected or manipulated.

Verbal consent was obtained from village leaders and participants before conducting household surveys. Written consent was not obtained from participants because of low literacy rates among local villages. Participants were informed about the survey, its purpose, and how the data would be used prior to consenting. As the surveys were anonymous (i.e., names of participants were not recorded), verbal consent was not recorded. This project was administered by WCS which does not have an Institutional Review Board for research ethics regarding social science surveys.

## Results

### Reef fish biomass

**Figure 2:**
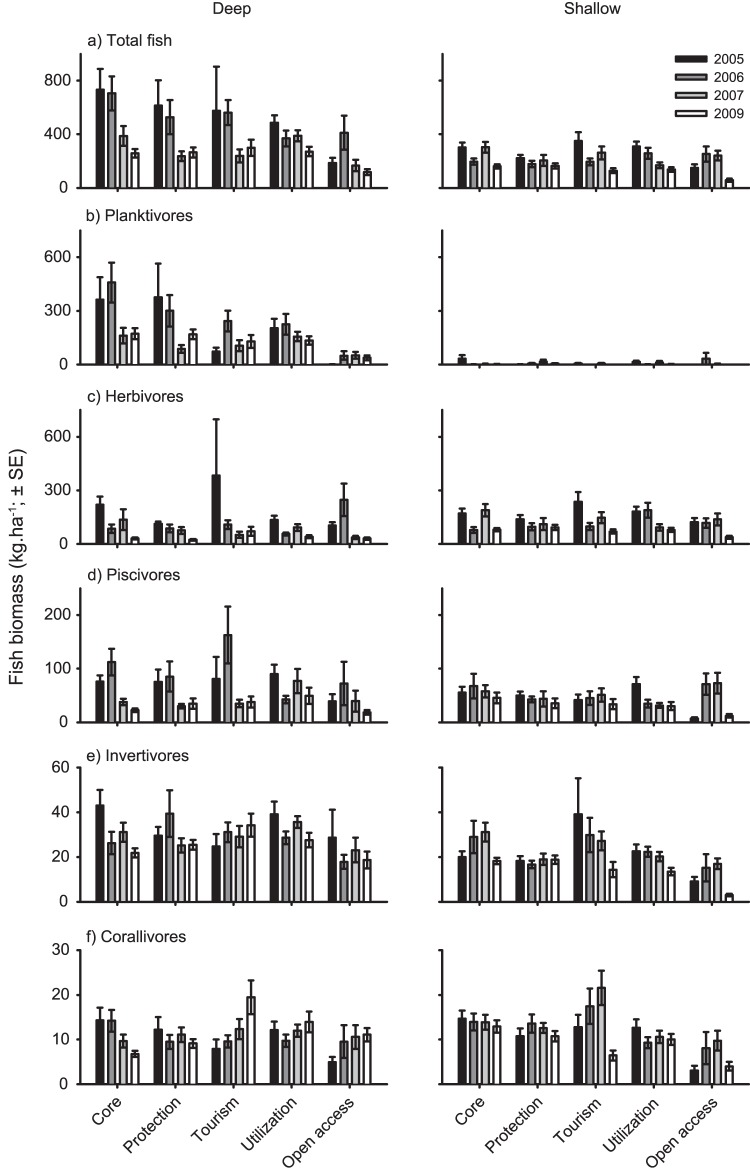
Changes in fish biomass following the 2005 rezoning of the Karimunjawa National Park. Variation in the biomass of (**A**) all fish collectively, (**B**) planktivorous fish, (**C**) herbivorous fish, (**D**) piscivorous fish, (**E**) invertivorous fish, and (**F**) corallivorous fish among management zones, depths and years.

Total reef fish biomass was generally greater in the deep habitat than in the shallow habitat, with annual estimates within each zone ranging from 118.4–733.4 kg.ha^−1^ and 57.4–349.9 kg.ha^−1^ in the deep and shallow habitats, respectively (Figure 2a). The deep fish assemblages were dominated by planktivores (45.7%) and herbivores (27.8%), while shallow water assemblages were dominated by herbivores (59.5%) and piscivores (21.8%). In the deep habitat, total fish biomass varied significantly among years and management zones (Table 2a). While overall fish biomass was lower outside the park at the open access sites than in the four management zones inside the park, there were marked declines in total fish biomass from 2005 to 2009 across all zones in the deep habitat (Table 2a, Figure 2a). These declines in overall fish biomass were primarily a result of decreases in the biomass of planktivores, herbivores, and, to a lesser extent, piscivores and invertivores in the deep habitats (Figure 2b–e). Corallivores showed varying responses, with biomass increasing within tourism zones and open access sites and decreasing within the core zone over the same time period (Figure 2f). There were small but significant declines in total fish biomass within the shallow habitat from 2005 to 2009 across all zones, although the magnitude of decline differed among zones (Table 2b; Figure 2a). These declines were largely driven by reductions in the biomass of herbivorous fishes (Figure 2c). Details of the Tukey's multiple comparisons are given in Table S3 and S4.

**Table 2:** Results of nested ANOVAs examining changes in fish and benthic composition following the 2005 rezoning of the Karimunjawa National Park.

	df	Total Fish	Planktivore	Herbivore	Piscivore	Invertivore	Coralivore	df	Coral	Algae
**A) Deep**										
Year	3	**17.466**	**3.226**	**42.683**	**20.295**	**3.287**	0.426	2	**39.154**	**79.928**
		**(<0.001)**	**(0.025)**	**(<0.001)**	**(<0.001)**	**(0.024)**	(0.735)		**(<0.001)**	**(<0.001)**
Management	4	**2.711**	**6.170**	0.364	2.000	2.159	0.640	4	0.437	0.384
		**(0.043)**	**(0.001)**	(0.833)	(0.112)	(0.090)	(0.637)		(0.781)	(0.818)
Year×Management	12	1.139	**2.198**	1.324	1.136	0.990	**2.456**	8	1.462	0.859
		(0.337)	**(0.017)**	(0.216)	(0.340)	(0.463)	**(0.007)**		(0.187)	(0.555)
Site (Management)	38	**2.214**	**2.631**	**2.183**	1.425	**1.650**	**1.690**	38	**8.326**	**5.135**
		**(<0.001)**	**(<0.001)**	**(0.001)**	(0.078)	**(0.023)**	**(0.018)**		**(<0.001)**	**(<0.001)**
Year×Site (Management)	109	**2.348**	**1.419**	1.040	**1.652**	**2.141**	**2.219**	71	**1.764**	**1.881**
		**(<0.001)**	**(0.013)**	(0.396)	**(0.001)**	**(<0.001)**	**(<0.001)**		**(<0.001)**	**(<0.001)**
Residual	257							392		
**B) Shallow**										
Year	3	**21.948**	**3.686**	**11.641**	**10.785**	**11.912**	**6.584**	2	**4.246**	**3.920**
		**(<0.001)**	**(0.014)**	**(<0.001)**	**(<0.001)**	**(<0.001)**	**(<0.001)**		**(0.018)**	**(0.025)**
Management	4	1.331	0.308	0.743	1.448	**10.100**	**5.298**	4	0.529	0.460
		(0.276)	(0.871)	(0.568)	(0.236)	**(<0.001)**	**(0.002)**		(0.714)	(0.765)
Year×Management	12	**2.272**	1.229	1.429	**3.164**	**2.044**	**2.085**	8	**2.513**	**2.928**
		**(0.013)**	(0.274)	(0.165)	**(0.001)**	**(0.028)**	**(0.024)**		**(0.019)**	**(0.008)**
Site (Management)	36	**2.204**	**1.862**	**2.212**	**1.947**	0.979	**2.141**	36	**7.228**	**6.984**
		**(0.001)**	**(0.007)**	**(0.001)**	**(0.004)**	(0.512)	**(0.001)**		**(<0.001)**	**(<0.001)**
Year×Site (Management)	103	**2.417**	**1.430**	**2.043**	**1.380**	**2.114**	**1.704**	66	**2.484**	**2.417**
		**(<0.001)**	**(0.013)**	**(<0.001)**	**(0.023)**	**(<0.001)**	**(<0.001)**		**(<0.001)**	**(<0.001)**
Residual	245							369		

Three factor nested ANOVAs comparing fish biomass and benthic cover among five management zones (core, protected, tourism, utilisation zones, and open access areas), four years (2005, 2006, 2007, 2009), and sites within (A) deep and (B) shallow habitats. F-ratios and p-values (in parentheses) are shown. Significant values (P<0.05) are given in bold.

### Benthic communities

**Figure 3:**
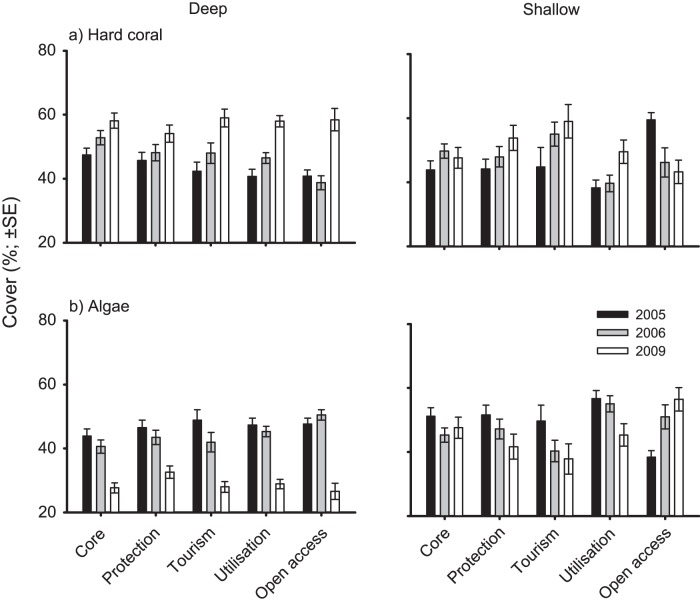
Temporal variation in benthic assemblages following the 2005 rezoning of the Karimunjawa National Park. Mean cover (±SE) of (**A**) hard coral and (**B**) macroalgae is given for four management zones within Karimunjawa National Park (Core, Protection, Tourism, and Utilisation) and open access areas outside the park boundaries.

Overall, mean coral cover varied from 38.8–60.0% and 38.2–59.5% in the deep and shallow habitats, respectively. Within the deep habitats coral cover increased significantly from 2005 to 2009 across all management zones and open access areas, with the rate of change varying among zones (Table 2a; Figure 3a). In contrast, temporal patterns of coral cover in shallow habitats varied markedly among zones. Coral cover increased significantly within protected, tourism, and utilisation zones, displayed limited change in core zones, yet decreased within open access areas outside the park boundaries (Table 2b, Figure 3a). Out of 10 areas surveyed (2 depths, 5 areas) habitat quality for reef fishes, as assessed via coral cover, improved in 8 of the areas and did not decline significantly in any area within KNP. No large rubble fields characteristic of blast fishing were observed during the visual censuses within the KNP.

The cover of algae varied from 26.6–50.5% in the deep and 37.9–56.7% in the shallow habitats, and displayed opposite patterns to that of live coral. From 2005 to 2009 algal cover decreased in all deep areas and within the shallow protected, tourism and utilisation zones, displayed limited variation in shallow core zones, and increased within shallow open access areas outside the park (Table 2; Figure 3b).

### Fishing pressure

**Figure 4:**
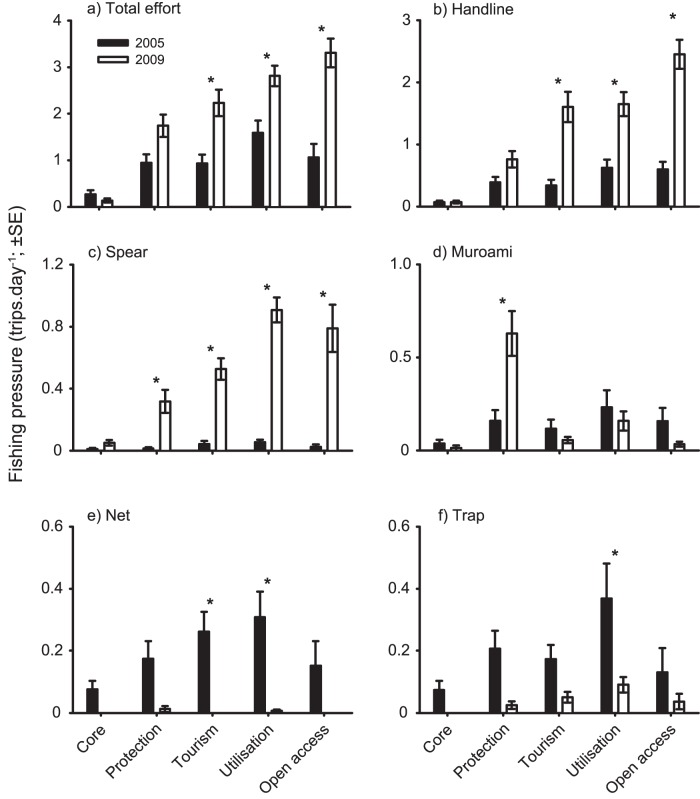
Changes in fishing pressure following the rezoning of the Karimanjawa National Park. Variation in total fishing effort and the use of five predominant fishing gears (handline, spear, muroami, nets, trap) within each management zones is compared between pre- and post-rezoning (i.e., 2005 and 2009, respectively). Asterisks indicate significant changes (p<0.05) in fishing pressure following the rezoning.

Landing site surveys indicated that total fishing impact (i.e., number of successful fishing trips pooled across all zones) approximately doubled between 2005 and 2009, increasing from 4.8 to 10.2 trips day^−1^, however the increases were not consistent among zones (zone×year: F_4,130_ = 6.73, P<0.001). Fishing impact increased significantly between 2005 and 2009 within tourism and utilisation zones and open access areas outside the park, while there were no significant changes in the core and protection zones (Figure 4a). Consequently, the proportion of total fishing impact within no-take zones (i.e., core and protection zones) decreased from 25.2% in 2005 to 18.4% in 2009. The use of different fishing gears also varied among years and zones in KNP (zone×year: Wilks λ = 0.33, F _20,419_ = 8.19, P<0.001). The use of handlines and spears increased significantly within tourism, utilisation, and open access zones between 2005 and 2009 (Figure 4b–c). The use of spears also increased within protection zones over the same period. In contrast, the use of small nets and traps tended to decrease, especially within the tourism and utilisation zones (Figure 4e–f). Although the use of poisons is not easily monitored (i.e., fishers often carry legitimate gears to mask cyanide fishing), qualitative information derived from discussions with local fishers indicated that the use of cyanide has declined considerably within the KNP during the study period. Fishers indicated that this reduction was related to declines in fishes targeted by cyanide (namely grouper), increased community support for bans on poisons (e.g., fishers caught using cyanide by local communities are handed over to law enforcement agencies), and the development of alternative livelihoods (i.e., grouper mariculture and tourism).

Interestingly, estimates of relative fishing effort from observed fishing trips displayed limited variation among the four management zones in 2009 (ANOVA: F_3,29_ = 1.60, P = 0.213), ranging from 2.1±0.6 (SE) to 4.1±2.8 trips km^−2^ day^−1^ in the tourism and utilisation zones, respectively (Figure S1).

### Awareness of park regulations, enforcement and resource dependency

Fishing is the most common occupation in and around KNP, accounting for 49.2% of the primary occupations. There was some variation in the dependency of fishing among communities, ranging from 54.2% in Karimunjawa, to 44.7 and 42.2% in Parang and Kemujan, respectively.

**Figure 5:**
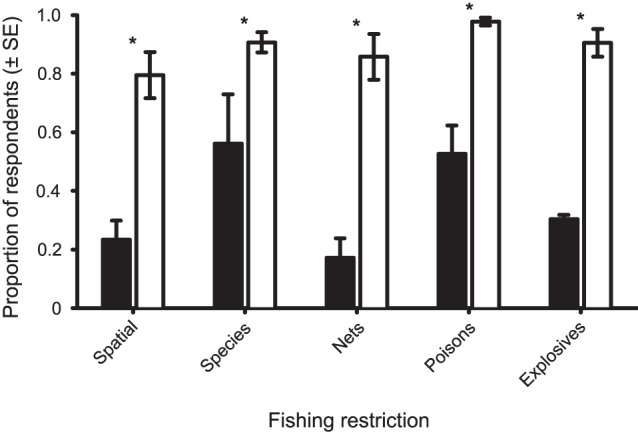
Changes in the awareness of fishing restrictions among fishers following the rezoning of the Karimunjawa National Park. The proportion of fishers from the three main fishing villages that were aware of the five main fishing restrictions prior to (2005) and four years after the rezoning (2009) of the Karimunjawa National Park. Asterisks indicate significant changes (p<0.05) in awareness following the rezoning.

Overall, the level of awareness of fishing restrictions was high among fishers from the major communities of Karimunjawa in 2009, ranging from 79.5±7.9 (SE) % for spatial closures to 97.7±1.2% for bans on the use of poisons. This represented a significant increase in the level of awareness from 2005 for all restriction types (Figure 5) for the three communities, both collectively and independently (Table S5).

Patrolling and enforcement was extremely limited in the KNP between 2005 and 2009 with a maximum of eight patrols per year (i.e., eight days per year), nearly all of which were close to the major communities of Karimunjawa and Kemujan. There were no prosecutions or fines imposed for violating spatial restrictions or for violations of any restrictions by members of the local communities. On three occasions fishers from outside the region (i.e., roving fishers) were prosecuted for violating gear restrictions after trawling over coral reef habitats.

## Discussion

Marine Protected Areas (MPAs) are widely advocated as a key tool for the effective conservation of coral reef resources (e.g., [12], [35]), with their effectiveness in conserving fish populations and habitat quality being largely dependent on the levels of compliance and enforcement [36], and the time since establishment [11], [37], [38]. The rezoning of the KNP appears to have had a positive effect on habitat quality. Coral cover increased across all zones within the park and decreased in shallow open access areas outside the park boundaries in the four years following the rezoning. There was, however, a marked decline in total fish biomass across all zones over the same period. These reductions were most pronounced in the deeper habitat, especially within the core and protection (i.e., no-take) zones where the biomass of piscivores, planktivores and herbivores declined by over 50% from 2005 to 2009.

Several studies have reported positive changes in reef fish biomass after four years of protection [11], [14], [37]–[39]. While the rate of recovery may vary with the state of the ecosystem and the depletion of fish biomass [36], the marked reductions in fish biomass indicate that the KNP no-take zones are not effectively protecting fish biomass. One plausible hypothesis to explain the lack of ecological response in the no-take zones at Karimunjawa is that a lack of compliance and/or enforcement is undermining the effectiveness of the KNP rezoning on fish communities. While compliance with bans on the use of destructive gears, such as bombs and cyanide, and a shift away from the use of small nets and traps may have contributed to the increased coral cover [40], [41], compliance with spatial restrictions appeared limited. Estimates of fishing intensity from direct observations of fishing vessels did not vary among zones, and the use of gears that target piscivorous (i.e., handlines and spears) and herbivorous fishes (i.e., spears) increased substantially from 2005 to 2009 across all but the core zones. The cause of the reduction in planktivorous fishes is not readily apparent, especially given the marked declines in the use of nets. However, caesionids (the dominant planktivores within the KNP) are captured in relatively large quantities by fishers using handlines and spears. This may, at least in part, have contributed to the decline of this group within the KNP. Despite considerable community consultation concerning the placement of the new zones in 2005, and a high level of awareness of spatial restrictions among local communities, compliance with the no-take zones was limited and has consequently undermined the capacity of the KNP to protect marine resources.

Fishing pressure is one of the greatest drivers of fish biomass in many coral reef systems (e.g., [42]–[44]), and appears to be primarily responsible for the declines in fish biomass within the KNP. Overall, total fishing impact doubled within the KNP from 2005 to 2009, driven largely by increases in the use of handlines and spears. Although our landing site surveys suggested that fishing impact was markedly lower within the core zones, direct observations of fishing vessels (i.e., observed fishing trips) failed to detect any difference in the distribution of effort among zones. This is not surprising given the former is an absolute measure (i.e., trips.day^−1^), while the later is a relative measure taking into account the size of the zones (i.e., trips.day^−1^.km^−2^). However, standardising the landing site data by the area of the respective zones had little influence on the estimates with fishing effort within core zones (0.017 trips.day^−1^.km^−2^) being 7-fold lower than those for protection and tourism zones (0.126 and 0.130 trips.day^−1^.km^−2^, respectively). The differences between these two estimates may reflect the reluctance of local fishers to self-report violations of spatial restrictions (i.e. stated compliance), or an increase in fishers from outside the KNP exploiting the local no-take zones, particularly the core zones. Furthermore, groups targeted by fisheries (i.e., piscivores, herbivores, and planktivores) declined markedly across all zones, while non-target groups (i.e., invertivores and corallivores) showed variable responses over time and among zones.

The observed declines in reef fish biomass within the KNP did not appear to be caused by changes in habitat quality. The physical and biological structure provided by hard coral and macroalgae are key determinants of fish communities on coral reefs, with the density, diversity and biomass of fish being generally positively related to coral cover and negatively related to macroalgal cover [45]–[50]. Although we did not quantify structural complexity per se, the increased coral and decreased macroalgal cover across all zones suggest that structural complexity is unlikely to have declined over the period of the study.

Changes in fishing effort and use of fishing gears may be influenced by a range of economic and social considerations, including resource dependency, gear profitability, access to markets, and regulatory controls [4], [51]. The reason for the observed shift from nets and traps towards spears and handlines is difficult to resolve but may be related to a combination of higher fuel prices influencing the relative profitability of the gears, the species targeted by the respective gears, relative catch per unit effort of the gears, and market demand for particular species or live fish.

Although KNP has legally prescribed spatial restrictions, patrolling and enforcement is limited. To our knowledge there have been no prosecutions for violating spatial restrictions within the KNP. Indeed, there are few legal precedents for prosecuting fishers using non-destructives gears within no-take areas in Indonesia. For example, within Komodo National Park extensive patrolling from three 16–20 m vessels (499 active patrol days in 2007) indicated that almost two-thirds of all boats were fishing in no-take zones, yet little is done to prosecute fishers for these violations [22]. Consequently fishing behaviour is largely driven by community needs and preferences regarding resource use [21]. Fishers are generally more willing to comply with gear restrictions than spatial restrictions, especially if profitability is maintained [51]–[54]. Within Indonesia species and gear restrictions are commonly enforced due to laws enacted in 1990 and 2004 [55] and the ease of meeting burden of proof requirements (i.e., the fish or gears can be confiscated, whereas proving a fisher was fishing in a certain area can be difficult). Furthermore, differences in the prescribed penalties for the use of destructive fishing gears or techniques (maximum 2 billion Rp fine and 5 year prison [56]) versus those for violations of spatial fishing restrictions (maximum 100 million Rp fine [57]), suggests that government agencies in Indonesia consider fishing inside no-take zones as less detrimental than destructive fishing. In marine settings the limited resources available for enforcement are often focused on minimising destructive fishing and protecting vulnerable or iconic species as opposed to increasing compliance with spatial restrictions [58].

Resource dependency on fishing is high within KNP and consequently food security may take priority over conservation goals [41]. It may be argued that this high dependency contributed to the lack of voluntary compliance with spatial restrictions, however there was a dichotomy in the compliance of spatial versus gear restrictions among local fishers. The awareness of both spatial and gear restriction was very high, and compliance with gear restrictions strong (i.e., explosives, poisons, and trawl nets), yet compliance with spatial restrictions was poor. The apparent willingness of the KNP communities to follow one set of operational rules but not another is difficult to explain. Discussions with local fishers and community members during the course of the study suggest that this dichotomy may be related to the perceived benefits of gear versus spatial restrictions. Local fishers appear to understand the rationale behind restrictions on the use of destructive gears (i.e., protecting the reef habitat and the links to fish communities) and are generally willing to comply with these restrictions. However, they see no immediate benefit in protecting fish in certain areas (i.e., no-take zones) if they are not to be made available to the community. External fishers exploiting no-take zones within the KNP may have also contributed to the lack of compliance with spatial restrictions (especially with the core zones), but they alone cannot account for the depletion of fish biomass within the no-take zones. Although stated compliance by KNP fishers, when standardized by the area of the respective zone, indicated the fishing impact was low in the core zones, there was considerable fishing by local communities within the no-take protection zones.

The increased and high awareness of spatial, species and gear restrictions following the rezoning is certainly a positive step. Future efforts need to focus on fostering the conditions that will facilitate greater compliance with no-take zones. For example, voluntary compliance with spatial restrictions may be enhanced if the no-take zones were located closer to the communities. Although greater distance to markets has been shown to enhance the effectiveness of customary management in some coral reef systems [4], [59], a recent study in Aceh, Indonesia suggested that proximity of a fishing location to a village assists in promoting compliance with fishing regulations [41]. Positioning no-take areas close to local communities has been shown to enhance stewardship and hence enforcement and compliance of spatial restrictions as fishing activities are in view of local communities [60], [61]. This was one of the recommendations of the 2005 KNP draft zoning plan, however following community consultation these zones were legislated considerable distances from the main villages. This decision reflected the needs of the local communities; presumably to allow all members of the community, not just those with boats, to gain access to reef resources for gleaning and fishing. Alternatively, co-management arrangements could be developed that essentially pass management of the resources to the communities themselves, allowing them to develop management systems along the lines of those that are proving successful in other areas of Indonesia [41], [62]. The KNP authority has coupled fisheries and conservation objectives to promote co-management that involves communities in both income generation and enforcement programs. The goal for programs such as this is to build fisheries biomass by reducing exploitation rates, reducing by-catch of non-target species and protecting habitat [63], [64].

### Conclusion

The results of the present study highlight the complexities of the social and economic dynamics that influence the ecological success of marine protected areas. Despite high awareness of spatial, species and gear restrictions, and strong compliance with gear restrictions among local fishers, compliance with spatial restrictions was poor and undermined the success of no-take areas in protecting fish communities. The mechanisms driving this dichotomy are unclear. Recent meta-analyses have demonstrated that human factors are stronger predictors of the ecological success of no-take reserves on coral reefs than physical design factors [36], [65]. The challenge now is to understand and promote the conditions that may improve compliance with spatial restrictions within KNP, and other marine protected areas throughout Indonesia.

## Supporting Information

Table S1 Co-ordinates of study sites within each management zone and number of replicate transects for the benthic and reef fish surveys.
(DOC)

Table S2 Checklist of fish species recorded and their trophic groupings in each of the management zones within KNP.
(DOC)

Table S3 Summary of Tukeys HSD multiple comparison tests to identify differences in fish and benthic communities among management zones and years in the deep habitat within Karimunjawa National Park (KNP). **(A)** Total fish biomass, **(B)** herbivorous fish biomass, **(C)** piscivorous fish biomass **(D)** invertivore biomass **(E)** corallivorous fish biomass, **(F)** planktivorous fish biomass, **(G)** coral cover, and **(H)** algal cover.
(DOC)

Table S4 Summary of Tukeys HSD multiple comparison tests to identify differences in fish and benthic communities among management zones and years in the shallow habitat within Karimunjawa National Park (KNP). **(A)** Planktivorous fish biomass, **(B)** herbivorous fish biomass, **(C)** total fish biomass **(D)** invertivore biomass **(E)** piscivorous fish biomass, **(F)** corallivorous fish biomass, **(G)** coral cover, and **(H)** algal cover.
(DOC)

Table S5 Summary of Chi-square tests comparing changes in the awareness of fishing restrictions before and after the 2005 rezoning of KNP. Chi-square and p-values are given for the three main villages independently and collectively.
(DOC)

Figure S1 **Comparison of direct observations of fishing effort among management zones within the Karimanjawa National Park.** The means are based on the number of boats observed fishing at 4–12 sites within each zone (Core: 4 sites; Protection: 8 sites; Tourism: 6 sites; Utilisation: 12 sites) during two days of each month in 2009.
(PDF)
