# Supplementary material for: Weak Compliance Undermines the Success of No-Take Zones in a Large Government-Controlled Marine Protected Area
Source: PLoS One. 2012 Nov 30;7(11):e50074. doi: 10.1371/journal.pone.0050074 (PMC3511441; doi:10.1371/journal.pone.0050074)
Supplement: Table S1 — Co-ordinates of study sites within each management zone and number of replicate transects for the benthic and reef fish surveys. (DOC) [file pone.0050074.s001.doc]

**Table S1 Co-ordinates of study sites within each management zone and number of replicate transects for the benthic and reef fish surveys.** Numbers in parentheses are the number of transects used for reef fish surveys. No benthic surveys were conducted in 2007.

|  | Latitude | Longitude | 2005 | | 2006 | | 2007 | | 2009 | |
| --- | --- | --- | --- | --- | --- | --- | --- | --- | --- | --- |
|  |  |  | Deep | Shallow | Deep | Shallow | Deep | Shallow | Deep | Shallow |
| **Core** |  |  |  |  |  |  |  |  |  |  |
| Kumbang 1 | 5.77121 S | 110.22629 E | 4 (2) | 4 (2) | 4 (2) | 4 (2) | (2) | (2) | 4 (4) | 4 (4) |
| Kumbang 2 | 5.77482 S | 110.22880 E | 4 (2) | 4 (2) | 4 (2) | 4 (2) | (2) | (2) | 4 (4) | 4 (4) |
| Kumbang 3 | 5.77514 S | 110.23345 E | 4 (2) | 4 (2) | 4 (2) | 4 (2) | (2) | (2) | 4 (4) | 4 (4) |
| Legon Janten | 5.86647 S | 110.46140 E | 4 (2) | 4 (2) | 4 (2) | 4 (2) | (2) | (2) | 4 (4) | 4 (4) |
| Legon Moto | 5.85771 S | 110.46753 E | 4 (2) | 4 (2) | 4 (2) | 4 (2) | (2) | (2) | 4 (4) | 4 (4) |
| Taka Malang E | 5.82247 S | 110.43879 E | 4 (2) | 4 (2) | 4 (2) | 4 (2) | (2) | (2) | 4 (4) | 4 (4) |
| Taka Malang W | 5.82071 S | 110.43885 E | 4 (2) | 4 (2) | 4 (2) | 4 (2) | (2) | (2) | 4 (4) | 4 (4) |
| Taka Menyawakan E | 5.76733 S | 110.32565 E | 4 (2) |  | 4 (2) |  | (2) |  | 4 (4) |  |
| Taka Menyawakan W | 5.76693 S | 110.32474 E | 4 (2) |  | 4 (2) |  | (2) |  | 4 (4) |  |
| Tanjung Dua | 5.85682 S | 110.47279 E | 4 (2) | 4 (2) | 4 (2) | 4 (2) | (2) | (2) | 4 (4) | 4 (4) |
| Tanjung Sekoci | 5.86286 S | 110.46648 E | 4 (2) | 4 (2) | 4 (2) | 4 (2) | (2) | (2) | 4 (4) | (4) |
|  |  |  |  |  |  |  |  |  |  |  |
| **Protection** |  |  |  |  |  |  |  |  |  |  |
| Burung | 5.89102 S | 110.34657 E | 4 (2) | 4 (2) | 4 (2) | 4 (2) | (2) | (2) | 4 (4) | 4 (4) |
| Cemara Kecil 1 | 5.83129 S | 110.38224 E | 4 (2) | 4 (2) | 4 (2) | 4 (2) | (2) | (2) | 4 (4) | 4 (4) |
| Cemara Kecil 2 | 5.83323 S | 110.38117 E | 4 (2) | 4 (2) | 4 (2) | 4 (2) | (2) | (2) | 4 (4) | 4 (4) |
| Gelean | 5.88023 S | 110.35940 E | 4 (2) | 4 (2) | 4 (2) | 4 (2) | (2) | (2) | 4 (4) | 4 (4) |
| Gosong Selikur 1 | 5.72927 S | 110.20448 E | 4 (2) | 4 (2) | 4 (2) | 4 (2) | (2) | (2) | 4 (4) | 4 (4) |
| Gosong Selikur 2 | 5.72706 S | 110.20765 E | 4 (2) | 4 (2) | 4 (2) | 4 (2) | (2) | (2) | 4 (4) | 4 (4) |
| Gosong Tengah | 5.79980 S | 110.51065 E | 4 (2) | 4 (2) | 4 (2) | 4 (2) | (2) | (2) | 4 (4) | 4 (4) |
| Katang 1 | 5.79844 S | 110.16611 E | 4 (2) | 4 (2) | 4 (2) | 4 (2) | (2) | (2) | 4 (4) | 4 (4) |
| Katang 2 | 5.80296 S | 110.16441 E | 4 (2) | 4 (2) | 4 (2) | 4 (2) | (2) | (2) | 4 (4) | 4 (4) |
| Sintok 1 | 5.78476 S | 110.51927 E | 4 (2) | 4 (2) | 4 (2) | 4 (2) | (2) | (2) | 4 (4) | 4 (4) |
| Sintok 2 | 5.78817 S | 110.51389 E | 4 (2) | 4 (2) | 4 (2) | 4 (2) | (2) | (2) | 4 (4) | 4 (4) |
|  |  |  |  |  |  |  |  |  |  |  |
| **Tourism** |  |  |  |  |  |  |  |  |  |  |
| Bengkoang | 5.74239 S | 110.41747 E |  |  | 4 (2) | 4 (2) | (2) | (2) | 4 (4) | 4 (4) |
| Karang Kapal | 5.89937 S | 110.24403 E |  |  | 4 (2) | 4 (2) | (2) | (2) | 4 (4) | 4 (4) |
| Kembar | 5.74358 S | 110.19561 E | 4 (2) | 4 (2) | 4 (2) | 4 (2) | (2) | (2) | 4 (4) | 4 (4) |
| Menjangan Besar | 5.89806 S | 110.43379 E |  |  | 4 (2) | 4 (2) | (2) | (2) | 4 (4) | 4 (4) |
| Menjangan Kecil | 5.90131 S | 110.40563 E |  |  | 4 (2) | 4 (2) | (2) | (2) | 4 (4) | 4 (4) |
| Menyawakan | 5.79817 S | 110.34733 E |  |  | 4 (2) | 4 (2) | (2) | (2) | 4 (4) | 4 (4) |
| Tengah | 5.80877 S | 110.51163 E | 4 (2) | 4 (2) | 4 (2) | 4 (2) | (2) | (2) | 4 (4) | 4 (4) |
|  |  |  |  |  |  |  |  |  |  |  |
| **Utilisation** |  |  |  |  |  |  |  |  |  |  |
| Batu Putih | 5.80503 S | 110.49186 E | 4 (2) | 4 (2) | 4 (2) | 4 (2) | (2) | (2) | 4 (4) | 4 (4) |
| Cemara Besar 1 | 5.80675 S | 110.37822 E | 4 (2) | 4 (2) | 4 (2) | 4 (2) | (2) | (2) | 4 (4) | 4 (4) |
| Cemara Besar 2 | 5.80310 S | 110.38098 E | 4 (2) | 4 (2) | 4 (2) | 4 (2) | (2) | (2) | 4 (4) | 4 (4) |
| Karang Katang | 5.80412 S | 110.14931 E | 4 (2) | 4 (2) | 4 (2) | 4 (2) | (2) | (2) | 4 (4) | 4 (4) |
| Kecil | 5.82037 S | 110.51187 E | 4 (2) | 4 (2) | 4 (2) | 4 (2) | (2) | (2) | 4 (4) | 4 (4) |
| Nyamuk | 5.82071 S | 110.20645 E | 4 (2) | 4 (2) | 4 (2) | 4 (2) | (2) | (2) | 4 (4) | 4 (4) |
| Pantai Nirwana | 5.88680 S | 110.45017 E | 4 (2) | 4 (2) | 4 (2) | 4 (2) | (2) | (2) | 4 (4) | 4 (4) |
| Parang 1 | 5.75527 S | 110.26043 E | 4 (2) | 4 (2) | 4 (2) | 4 (2) | (2) | (2) | 4 (4) | 4 (4) |
| Parang 2 | 5.76801 S | 110.25826 E | 4 (2) | 4 (2) | 4 (2) | 4 (2) | (2) | (2) | 4 (4) | 4 (4) |
| Tanjung Lemu | 5.83365 S | 110.48851 E | 4 (2) | 4 (2) | 4 (2) | 4 (2) | (2) | (2) | 4 (4) | 4 (4) |
|  |  |  |  |  |  |  |  |  |  |  |
| **Open access** |  |  |  |  |  |  |  |  |  |  |
| Cendekian | 5.80419 S | 110.55876 E | 4 (2) | 4 (2) | 4 (2) | 4 (2) | (2) | (2) | 4 (4) | 4 (4) |
| Genting 1 | 5.86211 S | 110.60538 E | 4 (2) | 4 (2) | 4 (2) | 4 (2) | (2) | (2) | 4 (4) | 4 (4) |
| Genting 2 | 5.83360 S | 110.60616 E | 4 (2) | 4 (2) | 4 (2) | 4 (2) | (2) | (2) | 4 (4) | 4 (4) |
| Genting 3 | 5.84098 S | 110.61131 E | 4 (2) | 4 (2) | 4 (2) | 4 (2) | (2) | (2) | 4 (4) | 4 (4) |
