# Supplementary material for: Weak Compliance Undermines the Success of No-Take Zones in a Large Government-Controlled Marine Protected Area
Source: PLoS One. 2012 Nov 30;7(11):e50074. doi: 10.1371/journal.pone.0050074 (PMC3511441; doi:10.1371/journal.pone.0050074)
Supplement: Table S2 — Checklist of fish species recorded and their trophic groupings in each of the management zones within KNP. (DOC) [file pone.0050074.s002.doc]

**Table S2 Checklist of fish species recorded and their trophic groupings in each of the management zones within KNP.** C: Coralivore; D: Detritivore; H: Herbivore; I: Benthic Invertivore; O: Omnivore; Pi: Piscivore; Pl: Planktivore.

|  |  | **Core** | | | | **Protection** | | | | **Tourism** | | | | **Utilisation** | | | | **Open Access** | | | |
| --- | --- | --- | --- | --- | --- | --- | --- | --- | --- | --- | --- | --- | --- | --- | --- | --- | --- | --- | --- | --- | --- |
|  |  | 2005 | 2006 | 2007 | 2009 | 2005 | 2006 | 2007 | 2009 | 2005 | 2006 | 2007 | 2009 | 2005 | 2006 | 2007 | 2009 | 2005 | 2006 | 2007 | 2009 |
| **ACANTHURIDAE** |  |  |  |  |  |  |  |  |  |  |  |  |  |  |  |  |  |  |  |  |  |
| *Acanthurus lineatus* | H | ***** | ***** | ***** | ***** | ***** | ***** | ***** | ***** |  | ***** |  | ***** | ***** | ***** | ***** | ***** |  | ***** |  | ***** |
| *Acanthurus spp* | H | ***** |  | ***** |  | ***** |  |  |  |  |  | ***** |  | ***** |  |  |  |  | ***** |  |  |
| *A. nigrofuscus* | H |  |  | ***** |  |  |  | ***** |  |  |  | ***** |  |  |  | ***** |  |  |  |  | ***** |
| *Ctenochaetus binotatus* | D | ***** |  |  | ***** |  |  |  |  |  |  |  |  |  |  |  |  |  |  |  |  |
| *C. striatus* | D | ***** | ***** | ***** | ***** | ***** | ***** | ***** | ***** | ***** | ***** | ***** | ***** | ***** | ***** | ***** | ***** |  | ***** | ***** | ***** |
| *Naso hexacanthus* | Pl |  |  | ***** |  |  |  |  |  |  |  |  |  |  |  |  |  |  |  |  |  |
| *N. lituratus* | H | ***** | ***** | ***** |  |  |  |  |  |  |  |  |  |  |  |  |  |  | ***** |  |  |
| *N. unicornis* | H | ***** |  |  |  |  | ***** |  |  |  |  |  |  |  |  |  |  |  |  |  |  |
| *N. vlamingii* | Pl |  |  |  |  |  |  |  |  |  |  |  |  | ***** |  |  |  |  |  |  |  |
| *Zebrasoma scopas* | H |  |  |  |  |  |  |  |  |  |  |  | ***** |  | ***** | ***** |  |  | ***** |  |  |
| **APOGONIDAE** |  |  |  |  |  |  |  |  |  |  |  |  |  |  |  |  |  |  |  |  |  |
| *Apogon bandanensis* | Pl | ***** | ***** | ***** |  |  | ***** |  |  |  |  | ***** |  | ***** | ***** | ***** |  |  |  |  |  |
| *A. compressus* | Pl | ***** | ***** | ***** | ***** | ***** | ***** | ***** | ***** | ***** | ***** | ***** | ***** | ***** | ***** | ***** | ***** |  | ***** | ***** | ***** |
| *A. cyanosoma* | Pl |  | ***** |  |  | ***** | ***** |  | ***** |  | ***** |  |  | ***** | ***** | ***** |  |  |  |  |  |
| *A. fragilis* | Pl |  |  |  | ***** |  |  |  |  |  |  |  | ***** |  |  |  |  |  |  |  |  |
| *A. fucata* | Pl |  |  |  | ***** |  |  |  | ***** |  |  |  |  |  |  |  | ***** |  |  |  |  |
| *A. parvulus* | Pl |  |  | ***** | ***** |  |  | ***** | ***** |  |  | ***** |  |  |  | ***** | ***** |  |  |  |  |
| *A. sealei* | Pl |  | ***** | ***** | ***** |  | ***** |  | ***** |  | ***** |  | ***** | ***** | ***** | ***** | ***** |  |  | ***** |  |
| *Archamia zosterophera* | Pl | ***** |  | ***** | ***** | ***** | ***** |  | ***** |  |  |  |  | ***** |  | ***** | ***** |  | ***** |  |  |
| *Cheilodipterus artus* | Pl |  | ***** | ***** | ***** |  |  | ***** | ***** |  | ***** | ***** | ***** |  |  | ***** | ***** |  |  |  | ***** |
| *C. intermedius* | Pl |  | ***** |  |  |  |  |  |  |  |  | ***** |  | ***** | ***** | ***** |  |  |  |  |  |
| *C. isostigmus* | Pl |  |  |  | ***** |  |  |  |  |  |  |  |  |  |  |  |  |  |  |  |  |
| *C. macrodon* | Pl |  |  | ***** | ***** |  |  | ***** | ***** |  |  |  | ***** |  |  |  | ***** |  |  |  |  |
| *C. quinquilineatus* | Pl | ***** |  | ***** | ***** | ***** |  | ***** | ***** | ***** |  | ***** | ***** | ***** |  | ***** | ***** |  |  |  |  |
| *Sphaeramia nematoptera* | Pl | ***** |  |  | ***** | ***** | ***** | ***** | ***** |  |  |  |  | ***** |  |  | ***** |  |  |  |  |
| **AULOSTOMIDAE** |  |  |  |  |  |  |  |  |  |  |  |  |  |  |  |  |  |  |  |  |  |
| *Aulostomus chinensis* | Pi |  | ***** |  |  |  |  |  |  |  |  |  |  |  |  |  |  |  |  |  |  |
| **BALISTIDAE** |  |  |  |  |  |  |  |  |  |  |  |  |  |  |  |  |  |  |  |  |  |
| *Balistapus undulatus* | O | ***** | ***** | ***** |  | ***** | ***** | ***** | ***** |  | ***** | ***** |  | ***** | ***** | ***** |  |  | ***** |  | ***** |
| *Sufflamen bursa* | O | ***** | ***** | ***** |  |  | ***** | ***** | ***** |  | ***** |  |  |  |  |  |  |  |  |  |  |
| *S. chrysoptera* | O | ***** |  |  | ***** | ***** |  |  |  |  |  |  |  | ***** |  | ***** |  |  |  | ***** |  |
| *S. fraenatus* | O |  | ***** | ***** |  | ***** |  |  |  |  |  |  |  |  |  | ***** |  |  |  |  |  |
| **CAESIONIDAE** |  |  |  |  |  |  |  |  |  |  |  |  |  |  |  |  |  |  |  |  |  |
| *Caesio caerulaurea* | Pl | ***** | ***** | ***** | ***** | ***** | ***** | ***** | ***** | ***** | ***** | ***** | ***** | ***** | ***** | ***** | ***** |  | ***** | ***** | ***** |
| *C. cuning* | Pl | ***** | ***** | ***** | ***** | ***** | ***** | ***** | ***** | ***** | ***** | ***** | ***** | ***** | ***** | ***** | ***** |  | ***** | ***** | ***** |
| *C. lunaris* | Pl |  |  | ***** |  |  |  |  | ***** |  |  |  |  |  |  |  |  |  |  | ***** |  |
| *C. teres* | Pl |  |  |  | ***** |  |  | ***** | ***** |  |  | ***** | ***** |  |  |  | ***** |  |  |  | ***** |
| *C. xanthonota* | Pl |  |  |  | ***** |  |  |  |  |  |  |  | ***** |  |  |  |  |  |  |  |  |
| *Pterocaesio chrysozona* | Pl |  |  |  | ***** |  |  |  | ***** |  |  |  | ***** |  |  |  | ***** |  |  |  | ***** |
| *P. marri* | Pl | ***** | ***** |  |  | ***** | ***** |  |  |  |  |  |  |  |  |  |  |  |  |  |  |
| *P. tile* | Pl |  | ***** | ***** |  |  | ***** |  | ***** |  |  |  | ***** |  | ***** | ***** |  |  |  |  | ***** |
| **CARANGIDAE** |  |  |  |  |  |  |  |  |  |  |  |  |  |  |  |  |  |  |  |  |  |
| *Atule mate* | Pi |  |  |  |  | ***** |  |  |  |  |  |  |  |  |  |  |  |  |  |  |  |
| *Carangoides bajad* | Pi |  | ***** |  |  |  |  |  |  |  |  |  |  | ***** |  |  |  |  |  |  |  |
| *C. ferdau* | Pi |  |  |  |  |  |  |  |  |  |  |  | ***** |  |  |  |  |  |  |  |  |
| *C. plagiotaenia* | Pi |  | ***** |  |  |  | ***** |  |  |  | ***** |  |  |  |  |  |  |  |  |  |  |
| *Elagatis bipinnulata* | Pi |  | ***** |  | ***** |  |  |  | ***** |  |  |  |  |  |  |  |  |  |  | ***** |  |
| **CENTRISCIDAE** |  |  |  |  |  |  |  |  |  |  |  |  |  |  |  |  |  |  |  |  |  |
| *Aeoliscus strigatus* | Pl | ***** | ***** | ***** | ***** |  | ***** |  | ***** |  | ***** | ***** | ***** | ***** | ***** | ***** | ***** |  |  |  |  |
| **CHAETODONTIDAE** |  |  |  |  |  |  |  |  |  |  |  |  |  |  |  |  |  |  |  |  |  |
| *Chaetodon adiergatos* | C | ***** | ***** | ***** |  | ***** | ***** | ***** | ***** | ***** |  |  |  |  | ***** | ***** |  |  | ***** | ***** |  |
| *C. auriga* | I |  |  |  | ***** |  | ***** |  |  |  | ***** |  |  | ***** |  | ***** | ***** |  | ***** |  |  |
| *C. baronessa* | C | ***** |  | ***** | ***** | ***** |  |  | ***** |  |  |  | ***** |  |  |  | ***** |  |  |  | ***** |
| *C. decussatus* | C |  |  |  |  |  |  |  | ***** |  |  |  |  |  |  |  |  |  |  |  |  |
| *C. ephippium* | I |  |  |  |  |  | ***** |  |  |  |  | ***** |  |  |  |  |  |  |  | ***** |  |
| *C. lineolatus* | I | ***** |  |  |  |  | ***** |  |  |  |  | ***** | ***** | ***** |  |  |  |  | ***** |  |  |
| *C. lunula* | C |  |  |  |  |  | ***** |  |  |  |  | ***** |  |  | ***** |  |  |  | ***** |  |  |
| *C. lunulatus* | C | ***** | ***** | ***** | ***** | ***** | ***** | ***** | ***** | ***** | ***** | ***** | ***** | ***** | ***** | ***** | ***** |  | ***** | ***** | ***** |
| *C. melanotus* | I | ***** | ***** | ***** |  | ***** | ***** | ***** | ***** |  | ***** |  |  | ***** | ***** |  | ***** |  | ***** | ***** |  |
| *C. ocellicaudus* | C | ***** |  |  |  | ***** |  |  |  |  |  |  |  | ***** |  |  |  |  |  |  | ***** |
| *C. octofasciatus* | C | ***** | ***** | ***** | ***** | ***** | ***** | ***** | ***** | ***** | ***** | ***** | ***** | ***** | ***** | ***** | ***** |  | ***** | ***** | ***** |
| *C. rafflesi* | I |  |  |  | ***** |  |  |  | ***** |  |  |  |  | ***** |  |  |  |  |  |  |  |
| *C. speculum* | C |  | ***** | ***** |  |  |  |  |  |  |  |  |  |  |  |  | ***** |  |  |  | ***** |
| *C. triangulum* | C | ***** | ***** | ***** | ***** | ***** | ***** | ***** | ***** | ***** | ***** | ***** | ***** | ***** | ***** | ***** | ***** |  | ***** | ***** |  |
| *C. trifascialis* | C | ***** | ***** |  | ***** | ***** | ***** | ***** | ***** | ***** | ***** | ***** | ***** | ***** | ***** |  | ***** |  | ***** | ***** | ***** |
| *C. trifasciatus* | C | ***** |  |  | ***** | ***** | ***** | ***** |  | ***** |  | ***** | ***** | ***** |  | ***** | ***** |  |  | ***** |  |
| *C. ulietensis* | I |  |  |  |  |  |  |  |  |  |  |  |  | ***** |  |  |  |  |  |  |  |
| *C. vagabundus* | I |  |  | ***** |  | ***** | ***** |  | ***** | ***** | ***** |  |  | ***** |  | ***** |  |  | ***** | ***** |  |
| *C. wiebeli* | C | ***** |  |  |  |  |  |  | ***** |  |  | ***** |  |  |  |  |  |  |  |  | ***** |
| *Chelmon rostratus* | I | ***** | ***** | ***** | ***** |  |  |  |  | ***** | ***** | ***** | ***** | ***** | ***** | ***** | ***** |  | ***** | ***** | ***** |
| *Coradion altivelis* | I |  |  |  | ***** | ***** | ***** |  |  |  |  |  | ***** |  |  |  | ***** |  |  |  |  |
| *C. chrysozonus* | I | ***** | ***** | ***** | ***** | ***** | ***** | ***** | ***** | ***** | ***** | ***** |  | ***** | ***** | ***** |  |  | ***** |  | ***** |
| *Heniochus acuminatus* | I | ***** | ***** |  |  |  |  |  | ***** |  |  |  |  |  | ***** |  |  |  |  |  |  |
| *H. chrysostomus* | I | ***** |  |  |  |  |  |  |  |  |  |  |  | ***** |  |  |  |  |  |  |  |
| *H. monoceros* | I |  |  |  |  |  |  |  |  | ***** |  |  |  |  |  |  |  |  |  |  |  |
| *H. singularis* | I | ***** | ***** |  |  |  |  | ***** |  |  |  |  |  | ***** | ***** |  |  |  |  |  |  |
| *H. varius* | I | ***** | ***** | ***** | ***** | ***** | ***** | ***** | ***** | ***** | ***** | ***** | ***** | ***** | ***** | ***** | ***** |  | ***** | ***** | ***** |
| **DIODONTIDAE** |  |  |  |  |  |  |  |  |  |  |  |  |  |  |  |  |  |  |  |  |  |
| *Diodon hystrix* | I |  | ***** | ***** |  |  | ***** |  | ***** |  |  |  | ***** | ***** |  |  |  |  |  |  | ***** |
| *D. liturosus* | I | ***** | ***** |  |  | ***** |  |  |  |  |  |  |  |  |  |  |  |  |  |  |  |
| **ECHENEIDAE** |  |  |  |  |  |  |  |  |  |  |  |  |  |  |  |  |  |  |  |  |  |
| *Echeneis naucrates* | I | ***** |  |  |  |  |  |  |  |  |  |  | ***** |  |  |  |  |  |  |  |  |
| **EPHIPPIDAE** |  |  |  |  |  |  |  |  |  |  |  |  |  |  |  |  |  |  |  |  |  |
| *Platax boersii* | H | ***** | ***** | ***** |  | ***** |  | ***** |  |  | ***** |  |  | ***** | ***** |  |  |  |  | ***** |  |
| *P. orbicularis* | H |  |  | ***** |  |  |  |  | ***** |  |  | ***** | ***** |  |  | ***** |  |  |  |  |  |
| *P. pinnatus* | H | ***** |  |  | ***** |  |  |  | ***** |  | ***** |  | ***** |  | ***** |  | ***** |  |  |  |  |
| *P. teira* | H |  | ***** | ***** | ***** | ***** |  | ***** | ***** |  | ***** | ***** |  | ***** | ***** | ***** |  |  | ***** |  |  |
| **HAEMULIDAE** |  |  |  |  |  |  |  |  |  |  |  |  |  |  |  |  |  |  |  |  |  |
| *Diagramma pictum* | I |  |  |  |  |  |  | ***** |  |  |  |  |  |  |  | ***** |  |  |  | ***** |  |
| *Plectorh. chaetodonoides* | I | ***** | ***** | ***** | ***** |  | ***** | ***** | ***** | ***** | ***** |  |  |  | ***** |  | ***** |  | ***** |  | ***** |
| *P. lessoni* | I |  |  |  |  |  |  |  |  |  |  |  |  |  |  | ***** |  |  |  |  |  |
| *P. picus* | I |  |  | ***** |  |  |  |  |  |  |  |  |  | ***** |  |  |  |  |  |  |  |
| **HOLOCENTRIDAE** |  |  |  |  |  |  |  |  |  |  |  |  |  |  |  |  |  |  |  |  |  |
| *Myripristis kuntee* | Pl |  |  |  |  |  | ***** |  | ***** |  |  |  |  |  |  |  | ***** |  |  |  |  |
| *M. murdjan* | Pl | ***** | ***** | ***** | ***** | ***** | ***** | ***** | ***** |  | ***** |  | ***** | ***** | ***** |  | ***** |  |  |  |  |
| *Neoniphon sammara* | Pl |  |  |  |  |  |  |  |  |  |  | ***** |  |  |  |  |  |  |  |  |  |
| *Sargocentrum cornutum* | Pl | ***** |  | ***** | ***** |  |  |  | ***** |  |  |  |  |  | ***** | ***** | ***** |  |  | ***** |  |
| *Sargocentrum spp* | Pl |  |  | ***** |  |  |  |  | ***** |  |  |  | ***** | ***** |  |  | ***** |  |  |  | ***** |
| **KYPHOSIDAE** |  |  |  |  |  |  |  |  |  |  |  |  |  |  |  |  |  |  |  |  |  |
| *Kyphosus bigibbus* | H |  | ***** |  |  |  |  |  | ***** |  |  |  | ***** |  | ***** |  |  |  |  |  |  |
| *K. vaigiensis* | H | ***** |  |  | ***** |  |  | ***** | ***** |  |  | ***** | ***** | ***** |  | ***** |  |  | ***** | ***** |  |
| **LABRIDAE (wrasses)** |  |  |  |  |  |  |  |  |  |  |  |  |  |  |  |  |  |  |  |  |  |
| *Anampses caeruleopunctatus* | I | ***** |  |  | ***** | ***** |  | ***** |  |  |  | ***** |  | ***** |  | ***** |  |  |  | ***** |  |
| *A. geographicus* | I | ***** |  | ***** |  |  |  |  |  |  |  |  |  |  |  | ***** | ***** |  |  |  |  |
| *Bodianius diana* | I |  |  |  |  |  |  |  |  |  |  |  |  | ***** |  |  |  |  |  |  |  |
| *B. mesothorax* | I | ***** | ***** | ***** | ***** | ***** |  |  | ***** | ***** | ***** | ***** | ***** | ***** | ***** | ***** | ***** |  |  | ***** |  |
| *Cheilinus chlorurus* | I | ***** | ***** |  | ***** | ***** | ***** |  | ***** | ***** | ***** |  | ***** | ***** | ***** |  | ***** |  | ***** |  | ***** |
| *C. fasciatus* | I | ***** | ***** | ***** | ***** | ***** | ***** | ***** | ***** | ***** | ***** | ***** | ***** | ***** | ***** | ***** | ***** |  | ***** | ***** | ***** |
| *C. oxycephalis* | I |  |  |  |  |  |  |  | ***** |  |  |  | ***** |  |  |  | ***** |  |  |  | ***** |
| *C. trilobatus* | I | ***** | ***** | ***** | ***** | ***** | ***** | ***** | ***** | ***** | ***** | ***** | ***** | ***** | ***** | ***** | ***** |  | ***** | ***** | ***** |
| *C. undulatus* | I |  |  | ***** |  |  |  | ***** |  |  |  |  |  |  | ***** |  | ***** |  |  |  |  |
| *Cheilio inermis* | Pi |  |  | ***** |  | ***** |  |  |  |  |  |  |  |  |  |  | ***** |  |  |  |  |
| *Choerodon anchorago* | I | ***** | ***** | ***** | ***** | ***** | ***** | ***** | ***** | ***** | ***** | ***** | ***** | ***** | ***** | ***** | ***** |  | ***** | ***** |  |
| *Cirrhilabrus cyanopleura* | Pl | ***** | ***** | ***** | ***** | ***** | ***** | ***** | ***** | ***** | ***** | ***** | ***** | ***** | ***** | ***** | ***** |  |  | ***** | ***** |
| *C. exquisitus* | Pl |  |  |  | ***** |  |  |  | ***** |  |  |  | ***** |  |  |  | ***** |  |  |  |  |
| *Coris batuensis* | I | ***** | ***** | ***** | ***** |  |  | ***** | ***** | ***** | ***** | ***** | ***** | ***** | ***** | ***** | ***** |  | ***** |  | ***** |
| *Diproctacanthus xanthurus* | C | ***** | ***** | ***** | ***** | ***** | ***** | ***** | ***** | ***** | ***** | ***** | ***** | ***** | ***** | ***** | ***** |  | ***** | ***** | ***** |
| *Epibulus insidiator* | Pi | ***** | ***** | ***** | ***** | ***** | ***** | ***** | ***** | ***** | ***** | ***** | ***** | ***** | ***** | ***** | ***** |  | ***** | ***** | ***** |
| *Gomphosus varius* | I |  |  |  | ***** |  |  | ***** |  |  |  |  |  | ***** | ***** |  | ***** |  |  |  | ***** |
| *Halichoeres biocellatus* | I |  |  |  |  |  |  |  |  |  |  |  | ***** |  |  |  |  |  |  |  |  |
| *H. chloropterus* | I | ***** | ***** | ***** | ***** | ***** | ***** | ***** | ***** | ***** | ***** | ***** | ***** | ***** | ***** | ***** | ***** |  |  | ***** |  |
| *H. chrysus* | I |  |  |  |  |  | ***** |  |  |  |  |  |  |  |  |  |  |  |  |  |  |
| *H. hortulanus* | I | ***** | ***** | ***** | ***** | ***** | ***** | ***** | ***** | ***** | ***** | ***** |  | ***** | ***** | ***** | ***** |  |  | ***** |  |
| *H. leucurus* | I | ***** | ***** | ***** | ***** | ***** | ***** | ***** | ***** | ***** | ***** | ***** | ***** | ***** | ***** | ***** | ***** |  | ***** | ***** | ***** |
| *H' margaritaceus* | I | ***** | ***** | ***** | ***** | ***** | ***** | ***** | ***** | ***** | ***** | ***** | ***** | ***** | ***** | ***** |  |  | ***** | ***** | ***** |
| *H. marginatus* | I | ***** | ***** | ***** | ***** | ***** | ***** | ***** | ***** | ***** | ***** | ***** | ***** | ***** | ***** | ***** | ***** |  | ***** | ***** | ***** |
| *H. melanochir* | I | ***** | ***** | ***** |  | ***** | ***** | ***** | ***** | ***** | ***** | ***** |  | ***** | ***** | ***** |  |  | ***** | ***** |  |
| *H. melanurus* | I | ***** | ***** | ***** | ***** | ***** | ***** | ***** | ***** | ***** | ***** | ***** | ***** | ***** | ***** | ***** | ***** |  | ***** |  |  |
| *H. prosopeion* | I | ***** |  |  | ***** | ***** |  | ***** | ***** |  |  |  | ***** | ***** |  | ***** | ***** |  |  |  | ***** |
| *H. purpurescens* | I | ***** |  | ***** |  |  |  | ***** |  |  |  | ***** | ***** |  |  | ***** |  |  |  |  |  |
| *H. richmondi* | I | ***** | ***** | ***** | ***** | ***** |  | ***** | ***** | ***** |  | ***** | ***** | ***** | ***** | ***** | ***** |  |  | ***** | ***** |
| *H. scapularis* | I | ***** | ***** |  | ***** | ***** | ***** |  | ***** | ***** |  | ***** | ***** | ***** | ***** |  | ***** |  | ***** |  |  |
| *H. vrolikii* | I | ***** | ***** | ***** | ***** | ***** | ***** | ***** | ***** | ***** | ***** | ***** | ***** | ***** | ***** | ***** | ***** |  | ***** | ***** | ***** |
| *Hemigymnus fasciatus* | I |  |  |  |  |  |  | ***** |  |  | ***** | ***** |  |  | ***** |  |  |  |  |  |  |
| *H. melapterus* | I | ***** | ***** | ***** | ***** | ***** | ***** | ***** | ***** | ***** | ***** | ***** | ***** | ***** | ***** | ***** | ***** |  | ***** | ***** | ***** |
| *Labrichthys unilineatus* | C | ***** | ***** | ***** | ***** | ***** | ***** | ***** | ***** | ***** | ***** | ***** | ***** | ***** | ***** | ***** | ***** |  | ***** | ***** | ***** |
| *Labroides dimidiatus* | I | ***** | ***** | ***** | ***** | ***** | ***** | ***** | ***** | ***** | ***** | ***** | ***** | ***** | ***** | ***** | ***** |  | ***** | ***** | ***** |
| *Oxycheilinus celebicus* | I | ***** | ***** | ***** | ***** | ***** | ***** | ***** | ***** | ***** | ***** | ***** | ***** | ***** | ***** | ***** | ***** |  | ***** |  |  |
| *O. diagrammus* | I | ***** | ***** | ***** | ***** | ***** | ***** | ***** | ***** | ***** | ***** | ***** | ***** | ***** | ***** | ***** | ***** |  | ***** | ***** | ***** |
| *Paracheilinus filamentosus* | Pl |  |  |  |  | ***** |  |  |  |  |  |  |  |  |  |  |  |  |  |  |  |
| *Pseudocheilinus hexataenia* | I |  |  |  |  | ***** |  |  | ***** |  |  |  |  |  |  |  |  |  |  |  |  |
| *Pseudodax mollucanus* | I |  |  |  | ***** |  |  |  |  |  |  |  |  |  |  |  |  |  |  |  |  |
| *Pteragogus cryptus* | I | ***** | ***** | ***** |  | ***** | ***** | ***** |  | ***** | ***** | ***** |  | ***** | ***** | ***** |  |  | ***** | ***** |  |
| *P. enneacanthus* | I |  |  |  |  | ***** |  |  |  |  |  |  |  |  |  |  |  |  |  |  |  |
| *Stethojulis bandanensis* | I | ***** |  | ***** |  |  |  |  | ***** |  | ***** |  |  |  | ***** | ***** |  |  | ***** |  |  |
| *S. strigiventer* | I |  |  | ***** | ***** |  |  | ***** | ***** |  |  |  | ***** |  |  | ***** | ***** |  |  | ***** | ***** |
| *S. trilineata* | I | ***** | ***** | ***** | ***** | ***** | ***** | ***** | ***** | ***** | ***** | ***** | ***** | ***** | ***** | ***** | ***** |  | ***** | ***** | ***** |
| *Thalassoma amblycephalum* | I |  |  |  |  | ***** |  |  | ***** |  |  |  | ***** | ***** |  |  | ***** |  |  |  | ***** |
| *T. hardwicki* | I |  |  | ***** | ***** | ***** | ***** | ***** | ***** |  | ***** | ***** | ***** |  | ***** | ***** | ***** |  | ***** | ***** | ***** |
| *T. janseni* | I |  | ***** | ***** |  |  | ***** |  |  |  | ***** | ***** |  | ***** | ***** |  |  |  |  |  |  |
| *T. lunare* | I | ***** | ***** | ***** | ***** | ***** | ***** | ***** | ***** | ***** | ***** | ***** | ***** | ***** | ***** | ***** | ***** |  | ***** | ***** | ***** |
| *T. lutescens* | I |  |  |  |  |  |  |  |  |  |  |  | ***** |  |  |  |  |  |  |  |  |
| **LABRIDAE (parrotfishes)** |  |  |  |  |  |  |  |  |  |  |  |  |  |  |  |  |  |  |  |  |  |
| *Bolbometopon muricatum* | H | ***** |  | ***** | ***** | ***** |  |  |  |  |  |  |  | ***** |  | ***** |  |  |  |  |  |
| *Cetoscarus ocellatus* | H | ***** | ***** | ***** | ***** | ***** | ***** | ***** | ***** | ***** | ***** |  | ***** | ***** | ***** | ***** | ***** |  | ***** | ***** | ***** |
| *Chlorurus bleekeri* | H | ***** | ***** | ***** | ***** | ***** | ***** |  | ***** | ***** | ***** |  | ***** | ***** |  | ***** | ***** |  |  | ***** | ***** |
| *C. bowersi* | H | ***** | ***** | ***** | ***** | ***** | ***** | ***** | ***** | ***** | ***** | ***** | ***** | ***** | ***** | ***** | ***** |  | ***** | ***** | ***** |
| *C. capistratoides* | H |  |  |  | ***** |  |  |  | ***** |  |  |  | ***** |  |  |  | ***** |  |  |  | ***** |
| *C. microrhinos* | H | ***** | ***** |  | ***** | ***** | ***** | ***** | ***** | ***** | ***** |  | ***** | ***** | ***** | ***** | ***** |  | ***** | ***** | ***** |
| *C. sordidus* | H | ***** | ***** | ***** | ***** | ***** | ***** | ***** | ***** | ***** | ***** | ***** | ***** | ***** | ***** | ***** | ***** |  | ***** | ***** | ***** |
| *C. strongylocephalus* | H |  |  |  |  |  |  | ***** | ***** |  |  | ***** | ***** |  |  | ***** | ***** |  |  | ***** |  |
| *C. troschelli* | H | ***** |  | ***** |  | ***** |  | ***** |  |  |  |  |  | ***** |  |  |  |  |  |  |  |
| *Hipposcarus spp* | H |  | ***** | ***** |  | ***** | ***** | ***** | ***** |  | ***** | ***** |  | ***** | ***** | ***** |  |  | ***** |  |  |
| *Scarus altipinnis* | H |  |  |  |  |  |  | ***** |  |  |  | ***** | ***** |  |  | ***** |  |  |  |  |  |
| *S. chameleon* | H |  |  |  | ***** |  |  |  | ***** |  |  |  |  |  |  |  |  |  |  |  |  |
| *S. dimidiatus* | H | ***** | ***** | ***** | ***** | ***** | ***** | ***** | ***** |  | ***** | ***** | ***** | ***** | ***** | ***** | ***** |  | ***** | ***** |  |
| *S. flavipectoralis* | H | ***** |  |  | ***** | ***** |  |  |  |  |  |  |  | ***** |  |  |  |  |  |  |  |
| *S. forsteni* | H |  |  |  | ***** |  |  | ***** | ***** |  |  |  |  |  |  |  |  |  |  | ***** |  |
| *S. frenatus* | H | ***** | ***** | ***** | ***** |  | ***** | ***** | ***** |  | ***** | ***** | ***** |  | ***** | ***** | ***** |  | ***** | ***** | ***** |
| *S. ghobban* | H | ***** | ***** | ***** | ***** | ***** | ***** | ***** | ***** | ***** | ***** | ***** | ***** | ***** | ***** | ***** | ***** |  | ***** | ***** |  |
| *S. globiceps* | H |  |  | ***** | ***** |  |  |  | ***** |  |  |  | ***** |  |  |  | ***** |  |  |  |  |
| *S. hypselopterus* | H |  |  |  |  |  |  |  | ***** |  |  |  |  |  |  |  |  |  |  |  |  |
| *S. niger* | H | ***** | ***** | ***** | ***** | ***** | ***** | ***** | ***** | ***** | ***** | ***** | ***** | ***** | ***** | ***** | ***** |  | ***** | ***** | ***** |
| *S. oviceps* | H |  |  |  |  |  |  |  |  |  |  |  |  |  |  | ***** |  |  |  |  |  |
| *S. prasiognathus* | H | ***** |  | ***** | ***** | ***** |  | ***** | ***** |  | ***** | ***** |  |  | ***** | ***** | ***** |  | ***** |  | ***** |
| *S. psittacus* | H |  |  | ***** |  |  |  | ***** |  |  |  | ***** |  |  |  | ***** |  |  |  | ***** |  |
| *S. quoyii* | H | ***** | ***** | ***** | ***** | ***** | ***** | ***** | ***** | ***** | ***** | ***** | ***** | ***** | ***** | ***** | ***** |  | ***** | ***** | ***** |
| *S. rivulatus* | H | ***** | ***** | ***** | ***** | ***** | ***** | ***** | ***** | ***** | ***** |  |  | ***** | ***** | ***** | ***** |  | ***** |  |  |
| *S. schlegeli* | H | ***** |  | ***** | ***** | ***** |  | ***** | ***** | ***** |  |  | ***** | ***** |  |  | ***** |  | ***** | ***** |  |
| *S. spinus* | H | ***** |  | ***** | ***** | ***** | ***** |  | ***** |  |  | ***** | ***** | ***** | ***** | ***** |  |  |  |  | ***** |
| *S. tricolor* | H |  |  |  | ***** |  |  |  | ***** |  |  |  | ***** | ***** |  |  | ***** |  |  |  |  |
| **LETHRINIDAE** |  |  |  |  |  |  |  |  |  |  |  |  |  |  |  |  |  |  |  |  |  |
| *Lethrinus erythracanthus* | I |  |  | ***** | ***** |  |  | ***** | ***** |  |  |  |  |  |  | ***** |  |  |  | ***** |  |
| *L. erythropterus* | I | ***** | ***** |  | ***** | ***** | ***** | ***** | ***** | ***** | ***** |  | ***** | ***** | ***** |  | ***** |  | ***** |  |  |
| *L. harak* | I |  | ***** |  | ***** |  | ***** |  | ***** |  |  | ***** |  |  |  |  |  |  | ***** |  |  |
| *L. lentjan* | I |  |  |  | ***** |  |  |  |  |  |  |  |  |  |  |  |  |  |  |  |  |
| *L. olivaceous* | Pi |  |  |  |  |  |  |  | ***** |  |  |  |  |  |  |  |  |  |  |  |  |
| *L. ornatus* | I |  | ***** |  |  |  |  |  |  |  | ***** |  |  |  | ***** |  |  |  |  |  |  |
| **LUTJANIDAE** |  |  |  |  |  |  |  |  |  |  |  |  |  |  |  |  |  |  |  |  |  |
| *Lutjanus argentimaculatus* | Pi |  |  |  |  |  |  |  |  |  |  |  |  | ***** |  |  |  |  |  |  |  |
| *L. biguttatus* | Pi |  | ***** | ***** | ***** | ***** | ***** | ***** | ***** | ***** | ***** | ***** | ***** | ***** | ***** | ***** | ***** |  | ***** | ***** |  |
| *L. bohar* | Pi |  | ***** |  |  |  |  |  |  |  |  |  |  | ***** |  |  |  |  |  |  |  |
| *L. carponotatus* | Pi | ***** | ***** | ***** | ***** | ***** | ***** | ***** |  | ***** | ***** | ***** |  | ***** | ***** | ***** | ***** |  |  |  |  |
| *L. decussatus* | Pi | ***** | ***** | ***** | ***** | ***** | ***** | ***** | ***** | ***** | ***** | ***** | ***** | ***** | ***** | ***** | ***** |  | ***** | ***** | ***** |
| *L. ehrenbergii* | Pi |  |  | ***** | ***** |  |  | ***** | ***** |  |  | ***** | ***** |  |  | ***** | ***** |  |  |  |  |
| *L. fulviflamma* | Pi | ***** | ***** |  | ***** | ***** | ***** | ***** | ***** |  | ***** |  | ***** | ***** | ***** |  | ***** |  | ***** | ***** |  |
| *L. kasmira* | Pi |  | ***** |  | ***** |  | ***** |  |  |  |  |  |  |  |  |  | ***** |  | ***** |  |  |
| *L. monostigma* | Pi |  |  |  |  |  |  |  |  |  |  |  |  | ***** |  |  |  |  |  |  |  |
| *L. quinquelineatus* | Pi | ***** |  |  | ***** | ***** |  |  |  |  |  |  |  | ***** |  |  |  |  |  |  |  |
| *L. sebae* | Pi |  | ***** |  |  |  |  |  |  |  |  |  |  |  |  |  |  |  |  |  |  |
| *Pinjalo pinjalo* | I |  |  |  | ***** |  |  |  |  |  |  |  |  |  |  |  |  |  |  |  |  |
| *Symphorichthys spilurus* | Pi |  | ***** |  |  |  |  |  |  |  |  |  |  |  |  |  |  |  |  |  |  |
| **MONACANTHIDAE** |  |  |  |  |  |  |  |  |  |  |  |  |  |  |  |  |  |  |  |  |  |
| *Aluterus scriptus* | O | ***** |  |  |  |  |  |  |  |  | ***** |  |  | ***** |  |  | ***** |  | ***** |  |  |
| *Amanses scopas* | I |  | ***** | ***** | ***** |  | ***** | ***** | ***** |  | ***** | ***** | ***** |  | ***** | ***** |  |  | ***** | ***** |  |
| *Cantherhines pardalis* | I |  |  |  |  |  |  |  |  | ***** |  |  |  | ***** |  |  |  |  |  |  |  |
| *Oxymonacanthus longirostris* | C |  |  |  |  | ***** | ***** |  | ***** |  | ***** |  |  | ***** | ***** |  | ***** |  |  | ***** |  |
| *Pervagor janthinosoma* | I |  |  |  |  |  |  |  |  |  |  |  |  |  |  |  | ***** |  |  |  |  |
| **MULLIDAE** |  |  |  |  |  |  |  |  |  |  |  |  |  |  |  |  |  |  |  |  |  |
| *Parupeneus barberinus* | I | ***** | ***** | ***** | ***** | ***** | ***** | ***** | ***** |  | ***** | ***** |  | ***** | ***** | ***** | ***** |  | ***** |  |  |
| *Upeneus tragula* | I | ***** |  | ***** | ***** | ***** |  | ***** | ***** |  |  |  | ***** |  |  | ***** |  |  |  |  |  |
| **MURAENIDAE** |  |  |  |  |  |  |  |  |  |  |  |  |  |  |  |  |  |  |  |  |  |
| *Gymnothorax javanicus* | Pi | ***** |  |  | ***** | ***** | ***** |  | ***** |  | ***** |  |  | ***** |  | ***** |  |  |  |  |  |
| **NEMIPTERIDAE** |  |  |  |  |  |  |  |  |  |  |  |  |  |  |  |  |  |  |  |  |  |
| *Pentapodus caninus* | I |  |  | ***** |  |  | ***** |  |  |  |  |  |  |  | ***** |  |  |  |  |  |  |
| *P. emeryi* | I |  |  |  |  |  |  |  | ***** |  |  |  |  | ***** |  |  |  |  |  |  |  |
| *P. trivitatus* | I |  |  | ***** | ***** | ***** |  | ***** | ***** |  |  | ***** |  | ***** | ***** | ***** | ***** |  |  |  |  |
| *Scolopsis bilineata* | I | ***** | ***** | ***** | ***** | ***** | ***** | ***** | ***** | ***** | ***** | ***** | ***** | ***** | ***** | ***** | ***** |  | ***** | ***** | ***** |
| *S. ciliatus* | I | ***** | ***** | ***** |  | ***** |  |  |  |  |  |  |  | ***** |  | ***** |  |  |  |  |  |
| *S. lineatus* | I | ***** | ***** | ***** | ***** | ***** | ***** | ***** | ***** | ***** | ***** | ***** | ***** | ***** | ***** | ***** | ***** |  | ***** | ***** | ***** |
| *S. margaritifer* | I | ***** | ***** | ***** | ***** | ***** | ***** | ***** | ***** | ***** | ***** | ***** | ***** | ***** | ***** | ***** | ***** |  | ***** | ***** | ***** |
| *S. monogramma* | I | ***** | ***** | ***** | ***** | ***** | ***** | ***** | ***** |  | ***** | ***** | ***** | ***** | ***** | ***** | ***** |  |  |  | ***** |
| *Scolopsis spp.* | I |  |  |  | ***** |  |  |  | ***** |  |  |  | ***** |  |  |  |  |  |  |  |  |
| **OSTRACIIDAE** |  |  |  |  |  |  |  |  |  |  |  |  |  |  |  |  |  |  |  |  |  |
| *Ostracion cubicus* | I | ***** |  | ***** |  | ***** | ***** | ***** |  |  |  |  | ***** | ***** | ***** |  |  |  |  |  |  |
| *O. meleagris* | I |  | ***** |  | ***** | ***** | ***** | ***** |  |  |  | ***** |  |  | ***** |  |  |  |  |  |  |
| **PEMPHERIDAE** |  |  |  |  |  |  |  |  |  |  |  |  |  |  |  |  |  |  |  |  |  |
| *Pempheris oualensis* | I |  | ***** |  |  |  |  |  |  |  |  |  |  |  |  |  |  |  |  |  |  |
| **POMACANTHIDAE** |  |  |  |  |  |  |  |  |  |  |  |  |  |  |  |  |  |  |  |  |  |
| *Centropyge tibicens* | H | ***** |  |  |  | ***** |  |  |  |  | ***** |  |  | ***** |  | ***** |  |  |  |  |  |
| *C. vroliki* | H | ***** |  |  |  | ***** | ***** |  |  |  |  | ***** | ***** |  |  |  |  |  |  |  |  |
| *Chaetodontoplus mesoleucus* | O | ***** | ***** | ***** | ***** | ***** | ***** | ***** | ***** | ***** | ***** | ***** | ***** | ***** | ***** | ***** | ***** |  | ***** | ***** | ***** |
| *Pomacanthus annularis* | O |  |  |  |  |  |  |  |  | ***** |  |  |  |  |  |  |  |  |  |  |  |
| *P.semicirculatus* | O |  |  |  |  |  |  |  |  |  |  |  |  |  | ***** |  |  |  |  |  |  |
| *P. sexstriatus* | O | ***** | ***** | ***** | ***** | ***** | ***** | ***** | ***** | ***** | ***** | ***** | ***** | ***** | ***** | ***** | ***** |  | ***** | ***** | ***** |
| *P. xanthometopon* | O |  |  |  |  |  |  |  |  |  |  |  |  |  |  |  |  |  |  |  | ***** |
| *Pygoplites diacanthus* | O | ***** | ***** | ***** | ***** | ***** | ***** | ***** | ***** | ***** | ***** | ***** | ***** | ***** | ***** | ***** | ***** |  | ***** | ***** | ***** |
| **PSEUDOCHROMIDAE** |  |  |  |  |  |  |  |  |  |  |  |  |  |  |  |  |  |  |  |  |  |
| *Pseudochromis fuscus* | Pi | ***** |  | ***** | ***** |  |  |  | ***** |  |  |  |  | ***** |  |  | ***** |  |  |  | ***** |
| **SERRANIDAE** |  |  |  |  |  |  |  |  |  |  |  |  |  |  |  |  |  |  |  |  |  |
| *Aetheloperca rogaa* | Pi |  |  |  |  |  |  |  |  |  | ***** |  |  |  |  |  |  |  |  |  |  |
| *Anyp. leucogrammiscus* | Pi | ***** | ***** | ***** | ***** | ***** | ***** | ***** | ***** |  | ***** | ***** | ***** | ***** |  | ***** | ***** |  |  |  | ***** |
| *Cephalopholis argus* | Pi | ***** | ***** | ***** | ***** | ***** | ***** | ***** | ***** | ***** | ***** | ***** | ***** | ***** | ***** | ***** |  |  | ***** | ***** | ***** |
| *C. boenak* | Pi |  |  | ***** | ***** |  | ***** | ***** | ***** |  |  | ***** | ***** |  |  | ***** | ***** |  |  |  | ***** |
| *C. cyanostigma* | Pi | ***** | ***** | ***** | ***** | ***** | ***** | ***** | ***** | ***** | ***** | ***** | ***** | ***** | ***** | ***** | ***** |  | ***** | ***** | ***** |
| *C. leopardus* | Pi | ***** |  |  | ***** | ***** | ***** |  | ***** |  |  |  | ***** |  |  | ***** | ***** |  |  |  |  |
| *C. microprion* | Pi | ***** |  | ***** | ***** | ***** |  | ***** | ***** | ***** |  | ***** | ***** | ***** |  | ***** | ***** |  |  |  | ***** |
| *C. miniata* | Pi |  |  |  |  | ***** |  |  |  |  |  |  |  |  |  |  |  |  | ***** |  |  |
| *C. urodeta* | Pi |  |  | ***** |  |  |  |  |  |  |  | ***** |  |  |  |  |  |  |  |  |  |
| *Diploprion bifasciatum* | Pi |  |  |  |  |  |  |  |  |  |  |  |  |  |  |  | ***** |  |  | ***** |  |
| *Epinephelus corallicolla* | Pi |  |  | ***** |  |  |  |  |  |  |  |  | ***** |  |  |  | ***** |  |  |  |  |
| *E. fasciatus* | Pi | ***** | ***** | ***** | ***** |  |  | ***** | ***** |  |  | ***** | ***** | ***** | ***** |  |  |  |  | ***** | ***** |
| *E. hexagonatus* | Pi |  |  | ***** |  |  |  |  |  |  |  |  |  |  |  |  |  |  |  |  |  |
| *E. macrospilos* | Pi |  |  |  |  |  |  |  |  |  |  |  | ***** |  |  |  |  |  |  |  |  |
| *E. merra* | Pi | ***** | ***** | ***** | ***** | ***** | ***** | ***** | ***** |  | ***** | ***** | ***** | ***** | ***** | ***** | ***** |  | ***** | ***** | ***** |
| *E. ongus* | Pi |  |  | ***** | ***** |  |  |  | ***** |  |  | ***** | ***** | ***** |  | ***** | ***** |  |  |  | ***** |
| *E. polyphekadion* | Pi |  |  |  |  |  | ***** |  |  |  |  |  |  |  |  | ***** |  |  |  |  |  |
| *E. quoyanus* | Pi |  | ***** |  | ***** |  | ***** |  |  |  |  |  | ***** |  |  |  |  |  | ***** |  |  |
| *Plectropomus areolatus* | Pi | ***** |  | ***** |  |  | ***** | ***** |  |  | ***** |  |  |  |  | ***** |  |  | ***** | ***** |  |
| *P. leopardus* | Pi |  |  |  |  |  | ***** |  |  |  |  |  |  |  |  |  |  |  | ***** |  |  |
| *P. maculatus* | Pi |  |  |  | ***** |  |  |  | ***** |  |  |  |  | ***** |  |  |  |  |  |  |  |
| *P. oligacanthus* | Pi | ***** | ***** | ***** | ***** |  | ***** | ***** | ***** |  |  |  |  | ***** | ***** | ***** | ***** |  |  |  |  |
| **SIGANIDAE** |  |  |  |  |  |  |  |  |  |  |  |  |  |  |  |  |  |  |  |  |  |
| *Siganus corallinus* | H | ***** | ***** | ***** | ***** | ***** | ***** | ***** | ***** | ***** | ***** |  | ***** | ***** | ***** | ***** | ***** |  | ***** | ***** | ***** |
| *S. doliatus* | H |  |  |  | ***** |  |  |  | ***** |  |  |  | ***** |  |  |  | ***** |  |  |  | ***** |
| *S. fuscescens* | H |  | ***** |  |  |  |  |  |  |  |  |  |  |  |  |  |  |  |  |  |  |
| *S. guttatus* | H | ***** | ***** | ***** | ***** | ***** | ***** | ***** | ***** |  | ***** | ***** | ***** | ***** | ***** | ***** | ***** |  | ***** | ***** | ***** |
| *S. javus* | H |  |  |  |  |  |  |  |  |  |  |  |  |  | ***** |  |  |  |  |  |  |
| *S. puelloides* | H |  |  |  | ***** |  |  | ***** | ***** |  |  |  |  |  |  |  | ***** |  |  | ***** |  |
| *S. puellus* | H | ***** | ***** | ***** | ***** | ***** | ***** | ***** | ***** | ***** | ***** | ***** | ***** | ***** | ***** | ***** | ***** |  |  |  |  |
| *S. punctatissimus* | H | ***** |  |  |  |  |  |  | ***** |  |  |  |  |  |  | ***** |  |  |  |  |  |
| *S. spinus* | H |  |  |  |  | ***** |  |  | ***** |  |  |  |  |  |  |  | ***** |  |  |  |  |
| *S. vermiculatus* | H |  |  |  |  |  | ***** |  |  |  |  |  |  |  |  | ***** |  |  |  |  |  |
| *S. virgatus* | H | ***** | ***** | ***** | ***** | ***** | ***** | ***** | ***** | ***** | ***** | ***** | ***** | ***** | ***** | ***** | ***** |  | ***** | ***** | ***** |
| *S. vulpinus* | H | ***** | ***** | ***** | ***** | ***** | ***** | ***** | ***** | ***** | ***** | ***** | ***** | ***** | ***** | ***** | ***** |  | ***** | ***** | ***** |
| **SPHYRAENIDAE** |  |  |  |  |  |  |  |  |  |  |  |  |  |  |  |  |  |  |  |  |  |
| *Sphyraenia flavicauda* | Pi |  | ***** |  | ***** | ***** |  | ***** | ***** | ***** | ***** |  |  | ***** |  |  | ***** |  |  | ***** |  |
| **SYNODONTIDAE** |  |  |  |  |  |  |  |  |  |  |  |  |  |  |  |  |  |  |  |  |  |
| *Saurida gracilis* | Pi |  |  |  |  |  |  |  | ***** |  |  |  | ***** |  |  |  | ***** |  |  |  | ***** |
| *Synodus vareigatus* | Pi | ***** | ***** | ***** | ***** | ***** | ***** | ***** |  | ***** | ***** |  | ***** |  | ***** | ***** | ***** |  |  | ***** |  |
| **TETRAODONTIDAE** |  |  |  |  |  |  |  |  |  |  |  |  |  |  |  |  |  |  |  |  |  |
| *Arothron nigropunctatus* | O | ***** | ***** | ***** | ***** | ***** | ***** | ***** | ***** |  | ***** | ***** | ***** | ***** | ***** | ***** | ***** |  | ***** |  | ***** |
| **ZANCLIDAE** |  |  |  |  |  |  |  |  |  |  |  |  |  |  |  |  |  |  |  |  |  |
| *Zanclus cornutus* | I | ***** | ***** |  | ***** | ***** | ***** |  | ***** | ***** |  |  |  | ***** |  | ***** | ***** |  |  | ***** |  |
