# Supplementary material for: Weak Compliance Undermines the Success of No-Take Zones in a Large Government-Controlled Marine Protected Area
Source: PLoS One. 2012 Nov 30;7(11):e50074. doi: 10.1371/journal.pone.0050074 (PMC3511441; doi:10.1371/journal.pone.0050074)
Supplement: Table S3 — Summary of Tukeys HSD multiple comparison tests to identify differences in fish and benthic communities among management zones and years in the deep habitat within Karimunjawa National Park (KNP). (A) Total fish biomass, (B) herbivorous fish biomass, (C) piscivorous fish biomass (D) invertivore biomass (E) corallivorous fish biomass, (F) planktivorous fish biomass, (G) coral cover, and (H) algal cover. (DOC) [file pone.0050074.s003.doc]

**Table S3 Summary of Tukeys HSD multiple comparison tests to identify differences in fish and benthic communities among management zones and years in the deep habitat within Karimunjawa National Park (KNP).** **(A)** Total fish biomass, **(B)** herbivorous fish biomass, **(C)** piscivorous fish biomass **(D)** invertivore biomass **(E)** corallivorous fish biomass, **(F)** planktivorous fish biomass, **(G)** coral cover, and **(H)** algal cover. Significant results (p < 0.05) are given in bold.

**(A)** Total fish biomass. Summary of Tukeys HSD multiple comparison tests to identify differences in the total fish biomass among years and management zones in KNP.

|  | 2005 | 2006 | 2007 |
| --- | --- | --- | --- |
| 2006 | 1.000 |  |  |
| 2007 | **< 0.001** | **< 0.001** |  |
| 2009 | **< 0.001** | **< 0.001** | **0.015** |

|  | Open Access | Utilisation | Tourism | Protected |
| --- | --- | --- | --- | --- |
| Utilization | **< 0.001** |  |  |  |
| Tourism | **< 0.001** | 0.567 |  |  |
| Protected | **< 0.001** | 0.396 | 1.000 |  |
| Core | **< 0.001** | 0.797 | 0.095 | **0.026** |

**(B)** Herbivorous fish biomass. Summary of Tukeys HSD multiple comparison tests to identify differences in the biomass of herbivorous fishes among years in KNP.

|  | 2005 | 2006 | 2007 |
| --- | --- | --- | --- |
| 2006 | **0.004** |  |  |
| 2007 | **< 0.001** | 0.240 |  |
| 2009 | **< 0.001** | **< 0.001** | **< 0.001** |

**(C)** Piscivorous fish biomass. Summary of Tukeys HSD multiple comparison tests to identify differences in the biomass of piscivorous fishes among years in KNP.

|  | 2005 | 2006 | 2007 |
| --- | --- | --- | --- |
| 2006 | 0.995 |  |  |
| 2007 | **0.005** | **0.002** |  |
| 2009 | **< 0.001** | **< 0.001** | **< 0.001** |

**(D)** Invertivore fish biomass. Summary of Tukeys HSD multiple comparison tests to identify differences in the biomass of invertebrate feeding fishes among years in KNP.

|  | 2005 | 2006 | 2007 |
| --- | --- | --- | --- |
| 2006 | 0.466 |  |  |
| 2007 | 0.781 | 0.955 |  |
| 2009 | **0.001** | 0.120 | **0.025** |

**(E)** Corallivorous fish biomass. Summary of Tukeys HSD multiple comparison tests to identify differences in the biomass of corallivorous fishes among management zones and years in KNP.

|  |  | Utilisation | | | | Tourism | | | | Protected | | | | Open Access | | | | Core | | |
| --- | --- | --- | --- | --- | --- | --- | --- | --- | --- | --- | --- | --- | --- | --- | --- | --- | --- | --- | --- | --- |
|  |  | 2005 | 2006 | 2007 | 2009 | 2005 | 2006 | 2007 | 2009 | 2005 | 2006 | 2007 | 2009 | 2005 | 2006 | 2007 | 2009 | 2005 | 2006 | 2007 |
| Utilisation | 2005 |  |  |  |  |  |  |  |  |  |  |  |  |  |  |  |  |  |  |  |
|  | 2006 | 1.000 |  |  |  |  |  |  |  |  |  |  |  |  |  |  |  |  |  |  |
|  | 2007 | 1.000 | 1.000 |  |  |  |  |  |  |  |  |  |  |  |  |  |  |  |  |  |
|  | 2009 | 1.000 | 1.000 | 1.000 |  |  |  |  |  |  |  |  |  |  |  |  |  |  |  |  |
| Tourism | 2005 | 1.000 | 1.000 | 1.000 | 1.000 |  |  |  |  |  |  |  |  |  |  |  |  |  |  |  |
|  | 2006 | 1.000 | 1.000 | 1.000 | 1.000 | 1.000 |  |  |  |  |  |  |  |  |  |  |  |  |  |  |
|  | 2007 | 1.000 | 1.000 | 1.000 | 1.000 | 1.000 | 1.000 |  |  |  |  |  |  |  |  |  |  |  |  |  |
|  | 2009 | 0.960 | 0.179 | 0.965 | 0.252 | 0.891 | 0.452 | 0.845 |  |  |  |  |  |  |  |  |  |  |  |  |
| Protected | 2005 | 1.000 | 1.000 | 1.000 | 1.000 | 1.000 | 1.000 | 1.000 | 0.704 |  |  |  |  |  |  |  |  |  |  |  |
|  | 2006 | 0.989 | 1.000 | 0.987 | 1.000 | 1.000 | 1.000 | 1.000 | **0.040** | 1.000 |  |  |  |  |  |  |  |  |  |  |
|  | 2007 | 1.000 | 1.000 | 1.000 | 1.000 | 1.000 | 1.000 | 1.000 | 0.534 | 1.000 | 1.000 |  |  |  |  |  |  |  |  |  |
|  | 2009 | 0.914 | 1.000 | 0.906 | 0.994 | 1.000 | 1.000 | 1.000 | 0.002 | 0.995 | 1.000 | 0.999 |  |  |  |  |  |  |  |  |
| Open Access | 2005 | **0.005** | 0.178 | **0.005** | **0.010** | 0.984 | 0.244 | 0.072 | **0.000** | **0.018** | 0.335 | **0.034** | 0.223 |  |  |  |  |  |  |  |
|  | 2006 | 0.998 | 1.000 | 0.998 | 1.000 | 1.000 | 1.000 | 1.000 | 0.383 | 1.000 | 1.000 | 1.000 | 1.000 | 0.880 |  |  |  |  |  |  |
|  | 2007 | 1.000 | 1.000 | 1.000 | 1.000 | 1.000 | 1.000 | 1.000 | 0.902 | 1.000 | 1.000 | 1.000 | 1.000 | 0.413 | 1.000 |  |  |  |  |  |
|  | 2009 | 1.000 | 1.000 | 1.000 | 1.000 | 1.000 | 1.000 | 1.000 | 0.961 | 1.000 | 0.998 | 1.000 | 0.980 | **0.016** | 1.000 | 1.000 |  |  |  |  |
| Core | 2005 | 1.000 | 0.955 | 1.000 | 0.996 | 0.999 | 0.993 | 1.000 | 1.000 | 1.000 | 0.751 | 0.999 | 0.386 | **0.000** | 0.951 | 1.000 | 1.000 |  |  |  |
|  | 2006 | 1.000 | 0.968 | 1.000 | 0.998 | 0.999 | 0.995 | 1.000 | 0.999 | 1.000 | 0.792 | 1.000 | 0.438 | 0.001 | 0.962 | 1.000 | 1.000 | 1.000 |  |  |
|  | 2007 | 0.997 | 1.000 | 0.997 | 1.000 | 1.000 | 1.000 | 1.000 | 0.076 | 1.000 | 1.000 | 1.000 | 1.000 | 0.238 | 1.000 | 1.000 | 1.000 | 0.863 | 0.892 |  |
|  | 2009 | 0.055 | 0.853 | 0.051 | 0.073 | 1.000 | 0.915 | 0.544 | **0.000** | 0.179 | 0.977 | 0.313 | 0.922 | 0.974 | 1.000 | 0.971 | 0.173 | **0.002** | **0.003** | 0.926 |

**(F)** Planktivorous fish biomass. Summary of Tukeys HSD multiple comparison tests to identify differences in the biomass of planktivorous fishes among management zones and years in KNP.

|  |  | Utilisation | | | | Tourism | | | | Protected | | | | Open Access | | | | Core | | |
| --- | --- | --- | --- | --- | --- | --- | --- | --- | --- | --- | --- | --- | --- | --- | --- | --- | --- | --- | --- | --- |
|  |  | 2005 | 2006 | 2007 | 2009 | 2005 | 2006 | 2007 | 2009 | 2005 | 2006 | 2007 | 2009 | 2005 | 2006 | 2007 | 2009 | 2005 | 2006 | 2007 |
| Utilisation | 2005 |  |  |  |  |  |  |  |  |  |  |  |  |  |  |  |  |  |  |  |
|  | 2006 | 1.000 |  |  |  |  |  |  |  |  |  |  |  |  |  |  |  |  |  |  |
|  | 2007 | 1.000 | 1.000 |  |  |  |  |  |  |  |  |  |  |  |  |  |  |  |  |  |
|  | 2009 | 0.980 | 0.569 | 0.999 |  |  |  |  |  |  |  |  |  |  |  |  |  |  |  |  |
| Tourism | 2005 | 1.000 | 1.000 | 1.000 | 1.000 |  |  |  |  |  |  |  |  |  |  |  |  |  |  |  |
|  | 2006 | 1.000 | 1.000 | 1.000 | 0.951 | 1.000 |  |  |  |  |  |  |  |  |  |  |  |  |  |  |
|  | 2007 | 0.962 | 0.620 | 0.993 | 1.000 | 1.000 | 0.919 |  |  |  |  |  |  |  |  |  |  |  |  |  |
|  | 2009 | 0.615 | 0.128 | 0.830 | 1.000 | 1.000 | 0.553 | 1.000 |  |  |  |  |  |  |  |  |  |  |  |  |
| Protected | 2005 | 1.000 | 0.994 | 1.000 | 1.000 | 1.000 | 1.000 | 1.000 | 0.984 |  |  |  |  |  |  |  |  |  |  |  |
|  | 2006 | 1.000 | 1.000 | 1.000 | 0.996 | 1.000 | 1.000 | 0.987 | 0.754 | 1.000 |  |  |  |  |  |  |  |  |  |  |
|  | 2007 | 0.950 | 0.514 | 0.992 | 1.000 | 1.000 | 0.902 | 1.000 | 1.000 | 1.000 | 0.984 |  |  |  |  |  |  |  |  |  |
|  | 2009 | 1.000 | 0.997 | 1.000 | 0.998 | 1.000 | 1.000 | 0.994 | 0.722 | 1.000 | 1.000 | 0.990 |  |  |  |  |  |  |  |  |
| Open Access | 2005 | **0.000** | **0.000** | **0.000** | **0.000** | **0.001** | **0.000** | **0.000** | **0.000** | **0.000** | **0.000** | **0.000** | **0.000** |  |  |  |  |  |  |  |
|  | 2006 | 0.319 | 0.080 | 0.477 | 0.943 | 0.997 | 0.263 | 0.999 | 1.000 | 0.738 | 0.417 | 0.995 | 0.436 | **0.019** |  |  |  |  |  |  |
|  | 2007 | 0.917 | 0.581 | 0.973 | 1.000 | 1.000 | 0.856 | 1.000 | 1.000 | 0.998 | 0.960 | 1.000 | 0.977 | **0.001** | 1.000 |  |  |  |  |  |
|  | 2009 | **0.006** | **0.000** | **0.016** | 0.173 | 0.936 | **0.007** | 0.873 | 0.844 | 0.063 | **0.010** | 0.606 | **0.004** | **0.008** | 1.000 | 0.999 |  |  |  |  |
| Core | 2005 | 1.000 | 1.000 | 1.000 | 0.775 | 1.000 | 1.000 | 0.787 | 0.239 | 1.000 | 1.000 | 0.709 | 1.000 | **0.000** | 0.137 | 0.731 | **0.001** |  |  |  |
|  | 2006 | 1.000 | 1.000 | 0.999 | 0.273 | 1.000 | 1.000 | 0.387 | 0.038 | 0.951 | 0.999 | 0.264 | 0.961 | **0.000** | **0.035** | 0.393 | **0.000** | 1.000 |  |  |
|  | 2007 | 1.000 | 1.000 | 1.000 | 1.000 | 1.000 | 1.000 | 0.998 | 0.906 | 1.000 | 1.000 | 0.998 | 1.000 | **0.000** | 0.566 | 0.988 | **0.024** | 1.000 | 0.993 |  |
|  | 2009 | 1.000 | 0.999 | 1.000 | 0.992 | 1.000 | 1.000 | 0.985 | 0.609 | 1.000 | 1.000 | 0.976 | 1.000 | **0.000** | 0.367 | 0.960 | **0.003** | 1.000 | 0.983 | 1.000 |

**(G)** Coral cover. Summary of Tukeys HSD multiple comparison tests to identify differences in the cover of scleractinian corals among years in KNP.

|  | 2005 | 2006 |
| --- | --- | --- |
| 2006 | **< 0.001** |  |
| 2009 | **< 0.001** | **< 0.001** |

**(H)** Algal cover. Summary of Tukeys HSD multiple comparison tests to identify differences in the cover of algae among years in KNP.

|  | 2005 | 2006 |
| --- | --- | --- |
| 2006 | **0.011** |  |
| 2009 | **< 0.001** | **< 0.001** |
