# Supplementary material for: Weak Compliance Undermines the Success of No-Take Zones in a Large Government-Controlled Marine Protected Area
Source: PLoS One. 2012 Nov 30;7(11):e50074. doi: 10.1371/journal.pone.0050074 (PMC3511441; doi:10.1371/journal.pone.0050074)
Supplement: Table S4 — Summary of Tukeys HSD multiple comparison tests to identify differences in fish and benthic communities among management zones and years in the shallow habitat within Karimunjawa National Park (KNP). (A) Planktivorous fish biomass, (B) herbivorous fish biomass, (C) total fish biomass (D) invertivore biomass (E) piscivorous fish biomass, (F) corallivorous fish biomass, (G) coral cover, and (H) algal cover. (DOC) [file pone.0050074.s004.doc]

**Table S4 Summary of Tukeys HSD multiple comparison tests to identify differences in fish and benthic communities among management zones and years in the shallow habitat within Karimunjawa National Park (KNP).** **(A)** Planktivorous fish biomass, **(B)** herbivorous fish biomass, **(C)** total fish biomass **(D)** invertivore biomass **(E)** piscivorous fish biomass, **(F)** corallivorous fish biomass, **(G)** coral cover, and **(H)** algal cover. Significant results (p < 0.05) are given in bold.

**(A) Planktivorous fish biomass. Summary of Tukeys HSD multiple comparison tests to identify differences in the total fish biomass among years in KNP.**

|  | 2005 | 2006 | 2007 |
| --- | --- | --- | --- |
| 2006 | 0.476 |  |  |
| 2007 | 0.366 | **0.010** |  |
| 2009 | 0.306 | 1.000 | **0.001** |

**(B)** Herbivorous fish biomass. Summary of Tukeys HSD multiple comparison tests to identify differences in the biomass of herbivorous fishes among years in KNP.

|  | 2005 | 2006 | 2007 |
| --- | --- | --- | --- |
| 2006 | **0.028** |  |  |
| 2007 | 0.104 | 0.956 |  |
| 2009 | **< 0.001** | **< 0.001** | **< 0.001** |

**(C)** Total fish biomass. Summary of Tukeys HSD multiple comparison tests to identify differences in total fish biomass among management zones and years in KNP.

|  |  | Utilisation | | | | Tourism | | | | Protected | | | | Open Access | | | | Core | | |
| --- | --- | --- | --- | --- | --- | --- | --- | --- | --- | --- | --- | --- | --- | --- | --- | --- | --- | --- | --- | --- |
|  |  | 2005 | 2006 | 2007 | 2009 | 2005 | 2006 | 2007 | 2009 | 2005 | 2006 | 2007 | 2009 | 2005 | 2006 | 2007 | 2009 | 2005 | 2006 | 2007 |
| Utilisation | 2005 |  |  |  |  |  |  |  |  |  |  |  |  |  |  |  |  |  |  |  |
|  | 2006 | 0.996 |  |  |  |  |  |  |  |  |  |  |  |  |  |  |  |  |  |  |
|  | 2007 | **0.018** | 0.687 |  |  |  |  |  |  |  |  |  |  |  |  |  |  |  |  |  |
|  | 2009 | **0.000** | **0.000** | 0.690 |  |  |  |  |  |  |  |  |  |  |  |  |  |  |  |  |
| Tourism | 2005 | 1.000 | 0.994 | 0.297 | **0.003** |  |  |  |  |  |  |  |  |  |  |  |  |  |  |  |
|  | 2006 | 0.647 | 1.000 | 1.000 | 0.112 | 0.839 |  |  |  |  |  |  |  |  |  |  |  |  |  |  |
|  | 2007 | 0.999 | 1.000 | 0.815 | **0.001** | 0.997 | 1.000 |  |  |  |  |  |  |  |  |  |  |  |  |  |
|  | 2009 | **0.000** | **0.001** | 0.861 | 1.000 | **0.006** | 0.227 | **0.005** |  |  |  |  |  |  |  |  |  |  |  |  |
| Protected | 2005 | 0.910 | 1.000 | 0.922 | **0.000** | 0.962 | 1.000 | 1.000 | **0.003** |  |  |  |  |  |  |  |  |  |  |  |
|  | 2006 | **0.032** | 0.819 | 1.000 | 0.405 | 0.378 | 1.000 | 0.907 | 0.643 | 0.974 |  |  |  |  |  |  |  |  |  |  |
|  | 2007 | **0.011** | 0.610 | 1.000 | 0.673 | 0.267 | 1.000 | 0.763 | 0.857 | 0.885 | 1.000 |  |  |  |  |  |  |  |  |  |
|  | 2009 | **0.000** | **0.001** | 0.967 | 1.000 | **0.012** | 0.372 | **0.008** | 1.000 | **0.005** | 0.838 | 0.966 |  |  |  |  |  |  |  |  |
| Open Access | 2005 | **0.024** | 0.601 | 1.000 | 0.995 | 0.225 | 0.999 | 0.717 | 0.999 | 0.850 | 1.000 | 1.000 | 1.000 |  |  |  |  |  |  |  |
|  | 2006 | 1.000 | 1.000 | 0.924 | **0.023** | 1.000 | 1.000 | 1.000 | **0.048** | 1.000 | 0.967 | 0.902 | 0.087 | 0.844 |  |  |  |  |  |  |
|  | 2007 | 1.000 | 1.000 | 0.927 | **0.024** | 0.999 | 1.000 | 1.000 | **0.049** | 1.000 | 0.968 | 0.905 | 0.090 | 0.849 | 1.000 |  |  |  |  |  |
|  | 2009 | **0.000** | **0.000** | **0.000** | **0.000** | **0.000** | **0.000** | **0.000** | **0.000** | **0.000** | **0.000** | **0.000** | **0.000** | **0.000** | **0.000** | **0.000** |  |  |  |  |
| Core | 2005 | 1.000 | 1.000 | 0.071 | **0.000** | 1.000 | 0.864 | 1.000 | **0.000** | 0.988 | 0.117 | **0.048** | **0.000** | 0.076 | 1.000 | 1.000 | **0.000** |  |  |  |
|  | 2006 | 0.540 | 1.000 | 1.000 | **0.037** | 0.821 | 1.000 | 1.000 | 0.105 | 1.000 | 1.000 | 1.000 | 0.186 | 0.997 | 1.000 | 1.000 | **0.000** | 0.805 |  |  |
|  | 2007 | 1.000 | 1.000 | 0.115 | **0.000** | 1.000 | 0.926 | 1.000 | **0.000** | 0.997 | 0.183 | 0.082 | **0.000** | 0.115 | 1.000 | 1.000 | **0.000** | 1.000 | 0.890 |  |
|  | 2009 | **0.000** | 0.160 | 1.000 | 0.767 | 0.122 | 0.990 | 0.342 | 0.933 | 0.425 | 1.000 | 1.000 | 0.992 | 1.000 | 0.652 | 0.659 | **0.000** | **0.003** | 0.967 | **0.006** |

**(D)** Invertivore biomass. Summary of Tukeys HSD multiple comparison tests to identify differences in the biomass of invertebrate feeding fishes among management zones and years in KNP.

|  |  | Utilisation | | | | Tourism | | | | Protected | | | | Open Access | | | | Core | | |
| --- | --- | --- | --- | --- | --- | --- | --- | --- | --- | --- | --- | --- | --- | --- | --- | --- | --- | --- | --- | --- |
|  |  | 2005 | 2006 | 2007 | 2009 | 2005 | 2006 | 2007 | 2009 | 2005 | 2006 | 2007 | 2009 | 2005 | 2006 | 2007 | 2009 | 2005 | 2006 | 2007 |
| Utilisation | 2005 |  |  |  |  |  |  |  |  |  |  |  |  |  |  |  |  |  |  |  |
|  | 2006 | 1.000 |  |  |  |  |  |  |  |  |  |  |  |  |  |  |  |  |  |  |
|  | 2007 | 1.000 | 1.000 |  |  |  |  |  |  |  |  |  |  |  |  |  |  |  |  |  |
|  | 2009 | **0.002** | **0.002** | **0.010** |  |  |  |  |  |  |  |  |  |  |  |  |  |  |  |  |
| Tourism | 2005 | 1.000 | 1.000 | 1.000 | 0.091 |  |  |  |  |  |  |  |  |  |  |  |  |  |  |  |
|  | 2006 | 1.000 | 1.000 | 1.000 | **0.000** | 1.000 |  |  |  |  |  |  |  |  |  |  |  |  |  |  |
|  | 2007 | 1.000 | 1.000 | 1.000 | **0.000** | 1.000 | 1.000 |  |  |  |  |  |  |  |  |  |  |  |  |  |
|  | 2009 | **0.043** | **0.043** | 0.129 | 1.000 | 0.226 | **0.004** | **0.004** |  |  |  |  |  |  |  |  |  |  |  |  |
| Protected | 2005 | 1.000 | 1.000 | 1.000 | 0.117 | 0.986 | 0.933 | 0.922 | 0.568 |  |  |  |  |  |  |  |  |  |  |  |
|  | 2006 | 0.998 | 0.998 | 1.000 | 0.284 | 0.961 | 0.806 | 0.785 | 0.807 | 1.000 |  |  |  |  |  |  |  |  |  |  |
|  | 2007 | 1.000 | 1.000 | 1.000 | 0.116 | 0.986 | 0.934 | 0.923 | 0.566 | 1.000 | 1.000 |  |  |  |  |  |  |  |  |  |
|  | 2009 | 0.960 | 0.959 | 0.997 | 0.123 | 0.901 | 0.469 | 0.441 | 0.720 | 1.000 | 1.000 | 1.000 |  |  |  |  |  |  |  |  |
| Open Access | 2005 | **0.002** | **0.002** | **0.006** | 1.000 | **0.025** | **0.000** | **0.000** | 0.989 | **0.050** | 0.112 | **0.050** | 0.074 |  |  |  |  |  |  |  |
|  | 2006 | 0.264 | 0.262 | 0.434 | 1.000 | 0.276 | 0.056 | 0.052 | 1.000 | 0.815 | 0.922 | 0.814 | 0.929 | 1.000 |  |  |  |  |  |  |
|  | 2007 | 1.000 | 1.000 | 1.000 | 0.808 | 0.996 | 0.994 | 0.993 | 0.973 | 1.000 | 1.000 | 1.000 | 1.000 | 0.396 | 0.970 |  |  |  |  |  |
|  | 2009 | **0.000** | **0.000** | **0.000** | **0.000** | **0.000** | **0.000** | **0.000** | **0.000** | **0.000** | **0.000** | **0.000** | **0.000** | **0.002** | **0.000** | **0.000** |  |  |  |  |
| Core | 2005 | 1.000 | 1.000 | 1.000 | 0.064 | 0.998 | 0.995 | 0.993 | 0.378 | 1.000 | 1.000 | 1.000 | 1.000 | **0.027** | 0.666 | 1.000 | **0.000** |  |  |  |
|  | 2006 | 1.000 | 1.000 | 1.000 | **0.001** | 1.000 | 1.000 | 1.000 | **0.018** | 0.998 | 0.981 | 0.998 | 0.838 | **0.001** | 0.158 | 1.000 | **0.000** | 1.000 |  |  |
|  | 2007 | 0.978 | 0.979 | 0.890 | **0.000** | 1.000 | 1.000 | 1.000 | **0.000** | 0.360 | 0.186 | 0.362 | **0.026** | **0.000** | **0.004** | 0.840 | **0.000** | 0.729 | 0.999 |  |
|  | 2009 | 1.000 | 1.000 | 1.000 | **0.018** | 0.984 | 0.889 | 0.872 | 0.286 | 1.000 | 1.000 | 1.000 | 1.000 | **0.017** | 0.709 | 1.000 | **0.000** | 1.000 | 0.996 | 0.218 |

**(E)** Piscivorous fish biomass. Summary of Tukeys HSD multiple comparison tests to identify differences in the biomass of piscivorous fishes among management zones and years in KNP.

|  |  | Utilisation | | | | Tourism | | | | Protected | | | | Open Access | | | | Core | | |
| --- | --- | --- | --- | --- | --- | --- | --- | --- | --- | --- | --- | --- | --- | --- | --- | --- | --- | --- | --- | --- |
|  |  | 2005 | 2006 | 2007 | 2009 | 2005 | 2006 | 2007 | 2009 | 2005 | 2006 | 2007 | 2009 | 2005 | 2006 | 2007 | 2009 | 2005 | 2006 | 2007 |
| Utilisation | 2005 |  |  |  |  |  |  |  |  |  |  |  |  |  |  |  |  |  |  |  |
|  | 2006 | 0.786 |  |  |  |  |  |  |  |  |  |  |  |  |  |  |  |  |  |  |
|  | 2007 | 0.915 | 1.000 |  |  |  |  |  |  |  |  |  |  |  |  |  |  |  |  |  |
|  | 2009 | **0.005** | 0.989 | 0.940 |  |  |  |  |  |  |  |  |  |  |  |  |  |  |  |  |
| Tourism | 2005 | 1.000 | 1.000 | 1.000 | 0.974 |  |  |  |  |  |  |  |  |  |  |  |  |  |  |  |
|  | 2006 | 0.999 | 1.000 | 1.000 | 0.806 | 1.000 |  |  |  |  |  |  |  |  |  |  |  |  |  |  |
|  | 2007 | 1.000 | 1.000 | 1.000 | 0.464 | 1.000 | 1.000 |  |  |  |  |  |  |  |  |  |  |  |  |  |
|  | 2009 | 0.319 | 1.000 | 1.000 | 1.000 | 1.000 | 1.000 | 0.991 |  |  |  |  |  |  |  |  |  |  |  |  |
| Protected | 2005 | 1.000 | 0.987 | 0.998 | **0.048** | 1.000 | 1.000 | 1.000 | 0.778 |  |  |  |  |  |  |  |  |  |  |  |
|  | 2006 | 1.000 | 0.998 | 1.000 | 0.106 | 1.000 | 1.000 | 1.000 | 0.904 | 1.000 |  |  |  |  |  |  |  |  |  |  |
|  | 2007 | 0.814 | 1.000 | 1.000 | 0.969 | 1.000 | 1.000 | 1.000 | 1.000 | 0.991 | 0.999 |  |  |  |  |  |  |  |  |  |
|  | 2009 | **0.024** | 1.000 | 0.997 | 1.000 | 0.995 | 0.963 | 0.758 | 1.000 | 0.172 | 0.314 | 0.999 |  |  |  |  |  |  |  |  |
| Open Access | 2005 | **0.000** | **0.001** | **0.000** | **0.019** | **0.024** | **0.000** | **0.000** | **0.001** | **0.000** | **0.000** | **0.000** | **0.003** |  |  |  |  |  |  |  |
|  | 2006 | 1.000 | 0.813 | 0.908 | 0.060 | 1.000 | 0.995 | 1.000 | 0.502 | 1.000 | 1.000 | 0.839 | 0.148 | **0.000** |  |  |  |  |  |  |
|  | 2007 | 1.000 | 0.996 | 0.999 | 0.401 | 1.000 | 1.000 | 1.000 | 0.944 | 1.000 | 1.000 | 0.997 | 0.644 | **0.000** | 1.000 |  |  |  |  |  |
|  | 2009 | **0.000** | **0.025** | **0.010** | 0.376 | 0.183 | **0.007** | **0.001** | **0.043** | **0.000** | **0.000** | **0.013** | 0.122 | 1.000 | **0.000** | **0.002** |  |  |  |  |
| Core | 2005 | 1.000 | 0.998 | 1.000 | 0.173 | 1.000 | 1.000 | 1.000 | 0.933 | 1.000 | 1.000 | 0.999 | 0.424 | **0.000** | 1.000 | 1.000 | 0.000 |  |  |  |
|  | 2006 | 1.000 | 0.996 | 1.000 | 0.124 | 1.000 | 1.000 | 1.000 | 0.889 | 1.000 | 1.000 | 0.998 | 0.335 | **0.000** | 1.000 | 1.000 | **0.000** | 1.000 |  |  |
|  | 2007 | 1.000 | 0.960 | 0.992 | **0.039** | 1.000 | 1.000 | 1.000 | 0.670 | 1.000 | 1.000 | 0.971 | 0.134 | **0.000** | 1.000 | 1.000 | **0.000** | 1.000 | 1.000 |  |
|  | 2009 | 0.339 | 1.000 | 1.000 | 0.993 | 1.000 | 1.000 | 0.995 | 1.000 | 0.815 | 0.930 | 1.000 | 1.000 | **0.000** | 0.546 | 0.962 | **0.014** | 0.954 | 0.918 | 0.711 |

**(F)** Corallivorous fish biomass. Summary of Tukeys HSD multiple comparison tests to identify differences in the biomass of corallivorous fishes among management zones and years in KNP.

|  |  | Utilisation | | | | Tourism | | | | Protected | | | | Open Access | | | | Core | | |
| --- | --- | --- | --- | --- | --- | --- | --- | --- | --- | --- | --- | --- | --- | --- | --- | --- | --- | --- | --- | --- |
|  |  | 2005 | 2006 | 2007 | 2009 | 2005 | 2006 | 2007 | 2009 | 2005 | 2006 | 2007 | 2009 | 2005 | 2006 | 2007 | 2009 | 2005 | 2006 | 2007 |
| Utilisation | 2005 |  |  |  |  |  |  |  |  |  |  |  |  |  |  |  |  |  |  |  |
|  | 2006 | 1.000 |  |  |  |  |  |  |  |  |  |  |  |  |  |  |  |  |  |  |
|  | 2007 | 1.000 | 1.000 |  |  |  |  |  |  |  |  |  |  |  |  |  |  |  |  |  |
|  | 2009 | 0.938 | 1.000 | 0.999 |  |  |  |  |  |  |  |  |  |  |  |  |  |  |  |  |
| Tourism | 2005 | 1.000 | 1.000 | 1.000 | 0.996 |  |  |  |  |  |  |  |  |  |  |  |  |  |  |  |
|  | 2006 | 1.000 | 0.962 | 1.000 | 0.586 | 1.000 |  |  |  |  |  |  |  |  |  |  |  |  |  |  |
|  | 2007 | 0.623 | 0.053 | 0.260 | **0.002** | 1.000 | 0.991 |  |  |  |  |  |  |  |  |  |  |  |  |  |
|  | 2009 | **0.018** | 0.538 | 0.125 | 0.628 | 0.525 | **0.004** | **0.000** |  |  |  |  |  |  |  |  |  |  |  |  |
| Protected | 2005 | 1.000 | 1.000 | 1.000 | 1.000 | 1.000 | 0.994 | 0.112 | 0.229 |  |  |  |  |  |  |  |  |  |  |  |
|  | 2006 | 1.000 | 0.960 | 1.000 | 0.456 | 1.000 | 1.000 | 0.921 | **0.001** | 0.995 |  |  |  |  |  |  |  |  |  |  |
|  | 2007 | 1.000 | 0.928 | 0.999 | 0.354 | 1.000 | 1.000 | 0.953 | **0.000** | 0.987 | 1.000 |  |  |  |  |  |  |  |  |  |
|  | 2009 | 1.000 | 1.000 | 1.000 | 1.000 | 1.000 | 0.963 | 0.024 | 0.075 | 1.000 | 0.951 | 0.906 |  |  |  |  |  |  |  |  |
| Open Access | 2005 | **0.000** | **0.000** | **0.000** | **0.000** | **0.005** | **0.000** | **0.000** | 0.221 | **0.000** | **0.000** | **0.000** | **0.000** |  |  |  |  |  |  |  |
|  | 2006 | 0.389 | 0.950 | 0.708 | 0.987 | 0.739 | 0.150 | **0.001** | 1.000 | 0.835 | 0.125 | 0.097 | 0.788 | 0.788 |  |  |  |  |  |  |
|  | 2007 | 1.000 | 1.000 | 1.000 | 1.000 | 1.000 | 1.000 | 0.515 | 0.872 | 1.000 | 1.000 | 1.000 | 1.000 | **0.006** | 0.977 |  |  |  |  |  |
|  | 2009 | **0.000** | **0.018** | **0.002** | **0.017** | 0.073 | **0.000** | **0.000** | 0.977 | **0.004** | **0.000** | **0.000** | **0.001** | 0.999 | 1.000 | 0.156 |  |  |  |  |
| Core | 2005 | 0.999 | 0.605 | 0.950 | 0.092 | 1.000 | 1.000 | 1.000 | **0.000** | 0.806 | 1.000 | 1.000 | 0.505 | **0.000** | **0.027** | 0.979 | **0.000** |  |  |  |
|  | 2006 | 1.000 | 0.963 | 1.000 | 0.521 | 1.000 | 1.000 | 0.962 | **0.002** | 0.995 | 1.000 | 1.000 | 0.959 | **0.000** | 0.137 | 1.000 | **0.000** | 1.000 |  |  |
|  | 2007 | 1.000 | 0.768 | 0.987 | 0.184 | 1.000 | 1.000 | 0.998 | **0.000** | 0.917 | 1.000 | 1.000 | 0.705 | **0.000** | **0.049** | 0.994 | **0.000** | 1.000 | 1.000 |  |
|  | 2009 | 1.000 | 0.973 | 1.000 | 0.387 | 1.000 | 1.000 | 0.696 | **0.000** | 0.998 | 1.000 | 1.000 | 0.958 | **0.000** | 0.128 | 1.000 | **0.000** | 1.000 | 1.000 | 1.000 |

**(G)** Coral cover. Summary of Tukeys HSD multiple comparison tests to identify differences in the cover of scleractinian corals among management zones and years in KNP.

|  |  | Core | | | Open access | | | Protection | | | Tourism | | | Utilisation | |
| --- | --- | --- | --- | --- | --- | --- | --- | --- | --- | --- | --- | --- | --- | --- | --- |
|  |  | 2005 | 2006 | 2009 | 2005 | 2006 | 2009 | 2005 | 2006 | 2009 | 2005 | 2006 | 2009 | 2005 | 2006 |
| Core | 2005 |  |  |  |  |  |  |  |  |  |  |  |  |  |  |
|  | 2006 | 0.828 |  |  |  |  |  |  |  |  |  |  |  |  |  |
|  | 2009 | 0.998 | 1.000 |  |  |  |  |  |  |  |  |  |  |  |  |
| Open access | 2005 | **0.000** | 0.097 | 0.012 |  |  |  |  |  |  |  |  |  |  |  |
|  | 2006 | 1.000 | 1.000 | 1.000 | **0.040** |  |  |  |  |  |  |  |  |  |  |
|  | 2009 | 1.000 | 0.958 | 1.000 | **0.004** | 1.000 |  |  |  |  |  |  |  |  |  |
| Protected | 2005 | 1.000 | 0.783 | 0.997 | **0.000** | 1.000 | 1.000 |  |  |  |  |  |  |  |  |
|  | 2006 | 0.987 | 1.000 | 1.000 | **0.007** | 1.000 | 0.998 | 0.981 |  |  |  |  |  |  |  |
|  | 2009 | 0.053 | 0.992 | 0.771 | 0.801 | 0.783 | 0.321 | **0.031** | 0.754 |  |  |  |  |  |  |
| Tourism | 2005 | 1.000 | 1.000 | 1.000 | 0.213 | 1.000 | 1.000 | 1.000 | 1.000 | 0.919 |  |  |  |  |  |
|  | 2006 | **0.037** | 0.944 | 0.588 | 0.994 | 0.611 | 0.207 | **0.024** | 0.570 | 1.000 | 0.814 |  |  |  |  |
|  | 2009 | **0.000** | **0.013** | **0.001** | 1.000 | **0.006** | **0.000** | **0.000** | **0.001** | 0.297 | 0.065 | 0.798 |  |  |  |
| Utilisation | 2005 | 0.820 | **0.007** | 0.124 | **0.000** | 0.742 | 0.982 | 0.749 | 0.036 | **0.000** | 0.988 | **0.000** | **0.000** |  |  |
|  | 2006 | 0.965 | 0.028 | 0.298 | **0.000** | 0.906 | 0.998 | 0.943 | 0.119 | **0.000** | 0.998 | **0.000** | **0.000** | 1.000 |  |
|  | 2009 | 0.843 | 1.000 | 1.000 | 0.060 | 1.000 | 0.965 | 0.798 | 1.000 | 0.983 | 1.000 | 0.908 | **0.007** | **0.007** | **0.026** |

**(H)** Algal cover. Summary of Tukeys HSD multiple comparison tests to identify differences in the cover of algae among management zones and years in KNP.

|  |  | Core | | | Open access | | | Protection | | | Tourism | | | Utilisation | |
| --- | --- | --- | --- | --- | --- | --- | --- | --- | --- | --- | --- | --- | --- | --- | --- |
|  |  | 2005 | 2006 | 2009 | 2005 | 2006 | 2009 | 2005 | 2006 | 2009 | 2005 | 2006 | 2009 | 2005 | 2006 |
| Core | 2005 |  |  |  |  |  |  |  |  |  |  |  |  |  |  |
|  | 2006 | 0.842 |  |  |  |  |  |  |  |  |  |  |  |  |  |
|  | 2009 | 0.999 | 1.000 |  |  |  |  |  |  |  |  |  |  |  |  |
| Open access | 2005 | **0.002** | 0.567 | 0.149 |  |  |  |  |  |  |  |  |  |  |  |
|  | 2006 | 1.000 | 0.982 | 1.000 | 0.069 |  |  |  |  |  |  |  |  |  |  |
|  | 2009 | 0.992 | 0.225 | 0.670 | **0.000** | 0.998 |  |  |  |  |  |  |  |  |  |
| Protected | 2005 | 1.000 | 0.661 | 0.991 | **0.000** | 1.000 | 0.997 |  |  |  |  |  |  |  |  |
|  | 2006 | 0.987 | 1.000 | 1.000 | 0.132 | 1.000 | 0.476 | 0.940 |  |  |  |  |  |  |  |
|  | 2009 | 0.067 | 0.995 | 0.786 | 0.998 | 0.452 | **0.008** | **0.019** | 0.802 |  |  |  |  |  |  |
| Tourism | 2005 | 1.000 | 1.000 | 1.000 | 0.636 | 1.000 | 0.997 | 1.000 | 1.000 | 0.960 |  |  |  |  |  |
|  | 2006 | **0.036** | 0.936 | 0.552 | 1.000 | 0.270 | **0.004** | **0.012** | 0.565 | 1.000 | 0.865 |  |  |  |  |
|  | 2009 | **0.000** | 0.058 | **0.007** | 0.998 | **0.004** | **0.000** | **0.000** | **0.004** | 0.591 | 0.188 | 0.967 |  |  |  |
| Utilisation | 2005 | 0.822 | **0.008** | 0.141 | **0.000** | 0.963 | 1.000 | 0.869 | **0.037** | **0.000** | 0.977 | **0.000** | **0.000** |  |  |
|  | 2006 | 0.988 | 0.053 | 0.443 | **0.000** | 0.998 | 1.000 | 0.995 | 0.190 | **0.000** | 0.998 | **0.000** | **0.000** | 1.000 |  |
|  | 2009 | 0.787 | 1.000 | 1.000 | 0.556 | 0.974 | 0.186 | 0.578 | 1.000 | 0.995 | 1.000 | 0.937 | 0.052 | **0.004** | **0.034** |
