# Supplementary material for: Weak Compliance Undermines the Success of No-Take Zones in a Large Government-Controlled Marine Protected Area
Source: PLoS One. 2012 Nov 30;7(11):e50074. doi: 10.1371/journal.pone.0050074 (PMC3511441; doi:10.1371/journal.pone.0050074)
Supplement: Table S5 — Summary of Chi-square tests comparing changes in the awareness of fishing restrictions before and after the 2005 rezoning of KNP. Chi-square and p-values are given for the three main villages independently and collectively. (DOC) [file pone.0050074.s005.doc]

**Table S3 Summary of Chi-square tests comparing changes in the awareness of fishing restrictions before and after the 2005 rezoning of KNP.** Chi-square and p-values are given for the three main villages independently and collectively.

| Restriction |  | Karimunjawa | Kemujan | Parang | TOTAL |
| --- | --- | --- | --- | --- | --- |
| Spatial | χ² | 30.52 | 26.35 | 31.10 | 84.03 |
|  | p | < 0.001 | < 0.001 | < 0.001 | < 0.001 |
| Species | χ² | 4.30 | 28.04 | 10.18 | 27.11 |
|  | p | 0.038 | < 0.001 | 0.001 | < 0.001 |
| Nets | χ² | 55.72 | 20.24 | 65.86 | 139.64 |
|  | p | < 0.001 | < 0.001 | < 0.001 | < 0.001 |
| Poisons | χ² | 19.15 | 18.44 | 37.99 | 69.44 |
|  | p | < 0.001 | < 0.001 | < 0.001 | < 0.001 |
| Explosives | χ² | 48.26 | 19.84 | 42.67 | 110.64 |
|  | p | < 0.001 | < 0.001 | < 0.001 | < 0.001 |
