# Supplementary figures and images for: Weak Compliance Undermines the Success of No-Take Zones in a Large Government-Controlled Marine Protected Area
Source: PLoS One. 2012 Nov 30;7(11):e50074. doi: 10.1371/journal.pone.0050074 (PMC3511441; doi:10.1371/journal.pone.0050074)

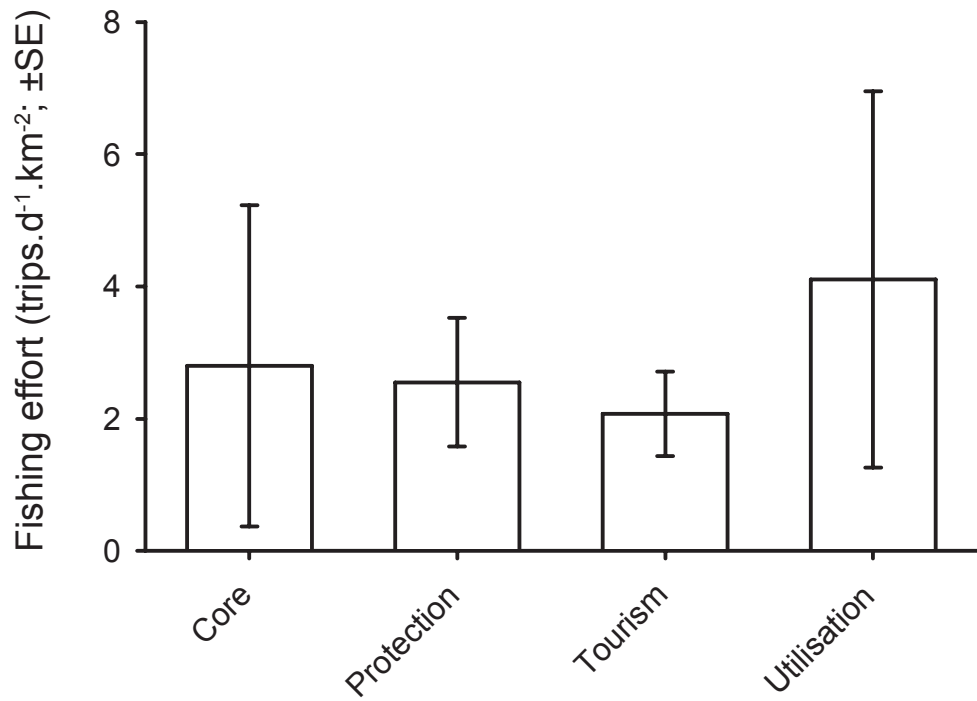

Supplement: Figure S1 — Comparison of direct observations of fishing effort among management zones within the Karimanjawa National Park. The means are based on the number of boats observed fishing at 4–12 sites within each zone (Core: 4 sites; Protection: 8 sites; Tourism: 6 sites; Utilisation: 12 sites) during two days of each month in 2009. (PDF) [file pone.0050074.s006.pdf]
